# Novel hypoxia-induced HIF-1αactivation in asthma pathogenesis

**DOI:** 10.1186/s12931-024-02869-0

**Published:** 2024-07-25

**Authors:** Mengzhi Wan, Qi Yu, Fei Xu, Lu Xia You, Xiao Liang, Kang kang Ren, Jing Zhou

**Affiliations:** Department of Respiratory Emergency and Critical Care Medicine, The First Affiliated Hospital of Nanchang University, No. 17, Yongwai Zheng Street, Nanchang, Jiangxi Province 330006 PR China

**Keywords:** Asthma, Hypoxia, HIF-1α, P53 ubiquitination, Airway Remodeling

## Abstract

**Background:**

Asthma’s complexity, marked by airway inflammation and remodeling, is influenced by hypoxic conditions. This study focuses on the role of Hypoxia-Inducible Factor-1 Alpha (HIF-1α) and P53 ubiquitination in asthma exacerbation.

**Methods:**

High-throughput sequencing and bioinformatics were used to identify genes associated with asthma progression, with an emphasis on GO and KEGG pathway analyses. An asthma mouse model was developed, and airway smooth muscle cells (ASMCs) were isolated to create an in vitro hypoxia model. Cell viability, proliferation, migration, and apoptosis were assessed, along with ELISA and Hematoxylin and Eosin (H&E) staining.

**Results:**

A notable increase in HIF-1α was observed in both in vivo and in vitro asthma models. HIF-1α upregulation enhanced ASMCs’ viability, proliferation, and migration, while reducing apoptosis, primarily via the promotion of P53 ubiquitination through MDM2. In vivo studies showed increased inflammatory cell infiltration and airway structural changes, which were mitigated by the inhibitor IDF-11,774.

**Conclusion:**

The study highlights the critical role of the HIF-1α-MDM2-P53 axis in asthma, suggesting its potential as a target for therapeutic interventions. The findings indicate that modulating this pathway could offer new avenues for treating the complex respiratory disorder of asthma.

**Supplementary Information:**

The online version contains supplementary material available at 10.1186/s12931-024-02869-0.

## Introduction

As a chronic inflammatory disease, asthma is characterized by recurrent symptoms such as wheezing, shortness of breath, and coughing [1]. The pathogenesis of this disease is complex, involving interactions between genes, environment, and immune factors [2]. In recent years, airway remodeling and airway inflammation associated with asthma have become hot research topics, as they are directly linked to the pathological and physiological processes and clinical manifestations of asthma [3]. Airway remodeling involves thickening of airway smooth muscle, hyperplasia of submucosal glands, and collagen deposition. These changes result in increased airway hyperresponsiveness, leading to increased severity and irreversibility of asthma [4]. Airway inflammation then promotes swelling and congestion of the airway wall, increasing mucus secretion and further exacerbating airway narrowing and obstruction [5].

Hypoxia is often observed in various illnesses, including asthma [6, 7]. Hypoxic conditions could induce changes in many cells and molecules, thus affecting the function of tissues and organs [8–10]. One of the key transcription factors induced by low oxygen is HIF-1α (hypoxia-inducible factor-1 alpha), which is upregulated under hypoxic conditions and plays a role in various biological processes such as cellular metabolism, proliferation, migration, and apoptosis [11–13]. Studies suggested that the inflammatory factor TNF-α affects the functionality of airway smooth muscle cells (ASMCs) and consequently the development of airway inflammatory diseases by regulating the protein and mRNA expression levels of HIF-1α [14]. However, the role of HIF-1α in airway inflammation induced by asthma and in the induction of airway remodeling remains unclear. Additionally, the specific molecular mechanisms through which HIF-1α regulates the vitality, proliferation, migration, and inflammatory response of ASMCs are not yet fully understood.

P53 is an important tumor suppressor protein in cell cycle regulation, DNA repair, and cell apoptosis processes [15]. Ubiquitination, as an essential protein degradation mechanism, could be facilitated by the action of MDM2 to promote ubiquitination of P53, thereby affecting the stability and activity of P53 [16]. Some studies suggest that the ubiquitination of P53 may be associated with airway remodeling and airway inflammation in asthma [17]. Recent research indicates that HIF-1α can suppress the expression of PPP1R1B, thereby inhibiting the degradation of p53 protein [9]. Furthermore, numerous studies have demonstrated a regulatory relationship between HIF-1α and p53 [18]. Nonetheless, the mechanisms by which HIF-1α influences the ubiquitination of P53 through regulating MDM2, and subsequently impacts airway remodeling and inflammation in asthma, remain unresolved [19].

Our research aims to explore how the upregulation of HIF-1α induced by low oxygen promotes P53 ubiquitination through the regulation of MDM2, thereby exacerbating airway inflammation in asthma and inducing airway remodeling through a series of in vitro and in vivo experiments. We have employed high-throughput sequencing, bioinformatics analysis, mouse models, and various cell biology techniques to delve into this mechanism [20]. This study is helpful in revealing the pathogenesis of asthma and may provide new targets and strategies for the diagnosis and treatment of this disease. Understanding this mechanism helps optimize treatment plans, improve patient management, and provide a foundation for future drug development and therapy. A better understanding of the roles of hypoxia, HIF-1α, MDM2, and P53 in asthma may contribute to developing more targeted and effective treatment strategies, leading to improved prognosis and quality of life for asthma patients.

## Materials and methods

### Construction of asthmatic mouse model

Purchase 45 healthy female BALB/c mice, aged 4–6 weeks and weighing 18–22 g, from the Shanghai Laboratory Animal Research Center. House each mouse separately in an SPF-grade animal laboratory. The laboratory humidity is 60-65%, the temperature is 25 ± 2℃, and free access to food and water is provided under alternating 12-hour light and dark conditions. The experiment starts one week after adapting to the feeding regimen, and the mice’s health status is observed before the experiment. The experimental protocol and animal usage plan have been approved by the Animal Ethics Committee [21].

Asthma was induced in mice by intraperitoneal injection of 20 µg ovalbumin (OVA, catalog number: 77,120, Thermo Scientific™, USA) and 1 mg aluminum hydroxide gel (catalog number: 1,017,502, Sigma-Aldrich, Germany) on days 0, 7, and 14. The injections were administered in 0.2 mL of sterile saline solution. Starting from day 21, the mice were placed in a custom-made airtight nebulization chamber and exposed to 1% OVA aerosol for 30 min daily for seven consecutive days. Before each inhalation, mice received a 0.2 mL intraperitoneal injection of saline solution prior to asthma induction. Mice in the control group were sensitized and challenged in the same manner but with saline replacing OVA. The procedure was consistent with the asthma group. Additionally, 0.2 mL of saline was intraperitoneally injected 30 min before each nebulization [22–24].

### Histological examination

The mouse lung tissue was fixed in a 4% paraformaldehyde solution and soaked for 24 h. Then, perform routine paraffin embedding to create consecutive sections of 4 μm. Using dichloromethane for dewaxing and then treating with alcohol of different concentrations (95%, 90%, and 85%), followed by washing with xylene for 10 min. Then, use hydrochloric acid alcohol for 5 s of coloration, followed by dehydration with alcohol of different concentrations (85%, 90%, 95%, 100%), and finally, transparent treatment with dichloromethane. Finally, use neutral resin to seal the specimen. The results were observed under an optical microscope (x 200, Olympus, Tokyo, Japan) [25].

### High-throughput sequencing of asthmatic mouse lung tissue samples

After establishing an asthma model in BALB/c mice, lung tissue samples were obtained from three mice in the Without asthma group and three mice in the Asthma group. Total RNA was extracted using a total RNA isolation kit (catalog number: 12,183,555, Invitrogen, USA) from the six samples, and the OD value of the total RNA was quantified. The integrity of these total RNA samples was evaluated using agarose gel electrophoresis. High-quality total RNA was reverse transcribed into cDNA to construct an RNA library, which was sequenced using Illumina’s NextSeq 500 platform. The raw imaging data obtained from sequencing was converted to raw reads through base calling. To ensure the quality of raw reads, cutadapt was utilized to remove sequencing adapter sequences and filter low-quality sequences, with the remaining reads referred to as “clean reads.” The sequences were aligned to the mouse reference genome using Hisat2 software, and gene expression was quantified using the R software package, resulting in a gene expression matrix [26].

### Bioinformatics analysis

Bioinformatics analysis of high-throughput sequencing data was performed using the “limma” package in R language to conduct differential expression screening of LncRNA and mRNA. The screening was based on a P-value < 0.05. Use the ggplot2 package to plot a volcano plot and the pheatmap package to plot a heatmap. Draw a Venn diagram using the Xiantao Academic Database.

Retrieve and download gene sets related to airway inflammation and airway remodeling in the GeneCards database (https://www.genecards.org/). Search and download gene sets related to airway inflammation using the PharmGKB database (https://www.pharmgkb.org/). Protein-protein interaction analysis was performed on candidate target gene-encoded proteins using the STRING database (https://cn.string-db.org/), and a protein interaction network graph was constructed using Cytoscape v3.10.0 software. Predicting the binding protein genes of HIF-1α using the BioGRID database (https://thebiogrid.org/) and HitPredict (http://www.hitpredict.org/). Perform KEGG and PANTHER analysis using the KOBAS database (http://bioinfo.org/kobas), and conduct pathway enrichment analysis and generate plots for both disease ontology (DO) and Gene Ontology (GO) using the “clusterProfiler”, “org.Hs.eg.db”, “enrichplot”, “DOSE”, and “ggplot2” packages in R language [27, 28].

### Isolation of airway smooth muscle cells (ASMC)

Euthanasia was performed on three healthy 8-week-old BALB/c wild-type mice using sodium pentobarbital (50 mg/kg BW). The mice were sensitized by intraperitoneal injection of 20 µg OVA and 2 mg aluminum hydroxide gel (dissolved in 200 µL PBS solution) on day 0 and day 14 and then challenged with 100 µg OVA solution (dissolved in 50 µL PBS) through intranasal administration on day 25–27. Under sterile conditions, the skin of mice was incised from the chest and abdomen to the jaw, then the sternum was incised upwards along the xiphoid process to expose the chest cavity. The heart and remaining tissues were removed, and the intact organs along with lung tissue were carefully extracted. The tissues were placed under a dissecting microscope, soaked in PBS containing 1% penicillin and streptomycin, and esophagus and connective tissues were carefully removed using fine scissors and forceps. The trachea was dissected from one side of the cartilaginous ring, and the smooth muscle tissue of the trachea with parallel striations in the middle was separated. Subsequently, the smooth muscle was cut into 1 mm^3^ pieces. The muscle pieces were then placed in a 2 mL PBS solution containing 1 mg/mL collagenase/neutral protease (Product Number: 10,269,638,001, Sigma-Aldrich, Germany) for a 30-minute digestion period. After digestion, the samples were centrifuged at 200 rpm for 5 min, the supernatant was removed, and they were washed with DMEM/F12 medium (Product Number: 30-2006, ATCC, USA) containing 10% FBS (Product Number: 10,099,141 C, Gibco, USA).

Culture the separated cells in Dulbecco’s Modified Eagle’s Medium (DMEM, Cat. No. 11,965,092, Gibco, USA) supplemented with 10% FBS, 1% penicillin, and streptomycin (Cat. No. 15,140,148, Gibco, USA). Place the cells in a humidified incubator (Heracell™ Vios 160i CR CO2 incubator, Cat. No. 51,033,770, Thermo Scientific™, Germany) at 37 ℃ and 5% CO2. After maturation, cells were passaged with 0.25% trypsin-0.02% EDTA solution (catalog number: 25,200,072, Gibco, USA). A typical feature of ASMCs is their growth in a valley-shaped form and identification of isolated ASMCs purity by immunofluorescent staining specific for smooth muscle-specific α-SMA [29].

### Construction and cultivation of in vitro cell models

The isolated mouse ASMCs were cultured on DMEM medium (Catalog number: 11,965,092, Gibco, USA), with the addition of 10% FBS (Catalog number: 10,099,141 C, Gibco, USA) and 1% penicillin-streptomycin (Catalog number: 15,140,148, Gibco, USA). Cells were cultured in a humidified incubator (Heracell™ Vios 160i CR CO2 incubator, catalog number: 51,033,770, Thermo Scientific™, Germany) at 37 ℃ and 5% CO2.

Upon reaching 80% confluence, airway smooth muscle cells (ASMCs) were subjected to passaging culturing. Cultures were maintained in a humidified incubator at 37 °C and 5% CO_2_, defining the conditions as normoxic (21% O2) culture. To establish a hypoxic environment (1% O_2_ or lower), cells from passages 3–8 were transferred to a cell hypoxia incubator (model: MIC-101, Billups Rothenberg, USA). A gas mixture of 0% O_2_, 5% CO_2_, and 95% N_2_ was introduced at a flow rate of 10 L/min for 15 min. Subsequently, the setup was sealed and placed in a 5% CO_2_, 37 °C incubator for 3 h to generate an in vitro hypoxia model of ASMCs. The achievement of hypoxia was confirmed by analyzing the PO_2_ levels in the cell culture medium using a blood gas analyzer (model: RAPIDLab 248, Siemens), ensuring a PO_2_ level below 35 mmHg. In comparison, the PO_2_ level in the supernatant of cells cultured under normoxic conditions was between 150 and 160 mmHg [30].

293T cells were purchased from ATCC (Catalog number: CRL-3216) and cultured in DMEM medium (Catalog number: 11,965,092, Gibco, USA) containing 10% FBS, 10 µg/mL streptomycin, and 100 U/mL penicillin. Cells were cultured in a humidified incubator (Heracell™ Vios 160i CR CO2 Incubator, catalog number: 51,033,770, Thermo Scientific™, Germany) at 37℃ with 5% CO2. A passaging culture should be performed when the cells grow to 80%~90% [31].

### Immunofluorescent staining

The test cells were plated in a 12-well cell culture plate for cell attachment one day in advance. After the cells are attached to the wall, discard the medium in the 12-well cell culture plate. Wash the cell culture in the well with DPBS (Catalog number: 14,040,133, Gibco, USA) 3 times. Fix cells with 4% paraformaldehyde (PFA) (Cat. No.: I28800, Thermo Scientific™, Germany) for 1 h. Wash the fixed cells twice with DPBS containing 0.05% Tween20 (catalog number 655,204, Sigma-Aldrich, Germany). Then, permeate with 0.1% Triton X-100 (Catalog Number: HFH10, Invitrogen, USA) for 3 min, followed by two washes with DPBS containing Tween20. Block samples in DPBS (blocking buffer) containing 5% goat serum (Catalog number: 16,210,072, Gibco, USA) and 0.3 M glycine (Catalog number: 50,046, Sigma-Aldrich, Germany) for 1 h. After this blocking step, the sample is incubated in a blocking buffer for 1 h, followed by adding primary antibody and overnight incubation at 4 °C.

The primary antibodies used in the experiment were purchased from Abcam (UK), including anti-α-SMA (catalog number: ab7817, 1 µg/mL). On the second day, rinse three times with Tween20-containing DPBS. Add sheep anti-mouse IgG (catalog number: A10551, 1:200) or sheep anti-rabbit (catalog number: A-11,008, 1:500) secondary antibody purchased from Invitrogen (USA), and incubate at room temperature for 1 h. After washing three times with DPBS, staining with Hoechst (Cat No: C0031, Solarbio, Beijing) for 5–10 min. Carefully remove the cell climbing slices from the cell culture plate using a bent fine injection needle and small forceps, and place them on glass slides coated with an anti-fluorescence quenching mounting medium (with the cell side facing down). Observed and photographed under a fluorescence microscope (FV-1000/ES, Olympus, Japan). The quantification method calculates the fluorescent coverage area under a 40x objective with a fixed field of view, and an average value is obtained by taking 6 fields of view per group [32, 33].

### Silent and overexpressed lentivirus vector construction

Based on GeneBank, potential short hairpin RNA (shRNA) target sequence analysis was performed on mouse cDNA sequences. First, three different sequences targeting HIF-1α and MDM2 were designed, and a sequence without any interfering sequence was used as the negative control (sh-NC). The primer sequences are shown in (Table S1), and the oligonucleotide was synthesized by GenePharma® (Shanghai, China). Constructed a lentiviral packaging system using panko.1 (a lentiviral gene silencing vector). The packaged virus and the intended vector were co-transfected into 293T cells using lipofectamine 2000 (with a cell confluence of 80–90%). The cell culture was collected after 48 h, and the supernatant was filtered and centrifuged to obtain viral particles. Collect the virus at the growth site and measure the virus titer through virus detection. The lentiviruses overexpressing HIF-1α were constructed and packaged by Genechem (Shanghai, China). The lentiviral gene overexpression vector was LV-PDGFRA [34–37].

### Transfection of cells

Digest the logarithmic growth phase cells with trypsin, resuspend in 5 × 10^4^ cells/mL and seed 2 mL per well in a 6-well plate. Before constructing the in vitro cell model, add various lentiviruses (MOI = 10, virus titer 1 × 10^8^ TU/mL) to the cell culture medium and incubate for 48 h. Screen stable cell lines using 2 µg/mL puromycin (catalog number: UC0E03, Sigma-Aldrich, Germany) for 2 weeks.

The cell transfection groups are as follows: (1) sh-NC group: transfected with negative control plasmid; (2) sh-HIF-1α group: transfected with sh-HIF-1α plasmid; (3) sh-MDM2 group: transfected with sh-MDM2 plasmid; (4) oe-NC group: transfected with oe-NC plasmid; (5) oe-HIF-1α group: transfected with oe-HIF-1α plasmid; (6) oe-MDM2 group: transfected with oe-MDM2 plasmid; (7) sh-NC + oe-NC group: co-transfected with sh-NC and oe-NC plasmids; (8) sh-HIF-1α + oe-NC group: co-transfected with sh-HIF-1α and oe-NC plasmids; (9) sh-HIF-1α + oe-MDM2 group: co-transfected with sh-HIF-1α and oe-MDM2 plasmids. After transfection for 48 h, RNA and protein levels were examined at 36 and 48 h, respectively, to validate the knockdown efficiency. The above plasmids were designed and synthesized by Guangzhou Ruibo Biotechnology Co., Ltd. (China) [38].

### RT-qPCR

Total RNA was extracted from tissue or cells using Trizol reagent (15,596,026, Invitrogen, USA). The concentration and purity of the total RNA were determined at 260/280 nm using NanoDrop LITE (catalog number: ND-LITE-PR, Thermo Scientific™, Germany). Synthesize cDNA from the extracted total RNA using PrimeScript RT reagent Kit with gDNA Eraser (Cat. No. RR047Q, TaKaRa, Japan).

Secondly, the SYBR Green PCR Master Mix (Catalog number: 4,364,344, Applied Biosystems, USA) and ABI PRISM 7500 Sequence Detection System (Applied Biosystems) were used to perform RT-qPCR detection of HIF-1α and various genes. The primers for HIF-1α and each gene were synthesized by TaKaRa company (Table S2), with GAPDH chosen as the reference gene. The relative expression levels of HIF-1α and each gene were analyzed using the 2^−ΔΔCt^ method, where ΔΔCt = (average Ct value of target gene in experimental group - average Ct value of housekeeping gene in experimental group) - (average Ct value of target gene in control group - average Ct value of housekeeping gene in control group). All RT-qPCR detections were performed in triplicate [39–41].

### Western blot

First, collect the organization or cells, then use enhanced RIPA lysis buffer containing protease inhibitors (catalog number: P0013B, Beyotime Biotechnology Co., Shanghai) for lysis. After that, use the BCA protein quantification kit (catalog number: P0012, Beyotime Biotechnology Co., Shanghai) to measure the protein concentration. 10% SDS-PAGE separated the protein sample, and the separated proteins were transferred to a PVDF membrane. The membrane was blocked with 5% BSA at room temperature for 2 h to block non-specific binding. After adding the diluted primary antibody, incubation was performed at room temperature for 1 h (details of the primary antibody are shown in Table S3). After washing the membrane, the membrane was incubated with HRP-labeled goat anti-rabbit secondary antibody (catalog number: ab6721, 1:2000, Abcam, UK) at room temperature for 1 h. Equal amounts of Solution A and B from the Pierce™ ECL western blot Substrate (catalog number: 32,209, Thermo Scientific™, Germany) were mixed in a dark room and then dropped onto the membrane. The membrane was exposed in a gel imaging system. Photography was performed using the Bio-Rad Imaging System (BIO-RAD, USA), and the Image J analysis software was used for grayscale quantification of each band in the Western blot images, with GAPDH as the internal reference. Each experiment is repeated three times [40].

### ELISA

The cells to be tested were seeded in a 96-well cell culture plate at a density of 3–5 × 10^4^ cells/mL and cultured under normal conditions for 48 h. Cells in the hypoxia-related treatment group were subjected to a gas mixture of 0% O_2_, 5% CO_2_, and 95% N_2_ at a flow rate of 10 L/min for 15 min. Subsequently, the setup was sealed and placed in a 5% CO_2_, 37 °C incubator for 3 h. Following the manufacturer’s instructions, levels of inflammatory cytokines in mouse bronchoalveolar lavage fluid (BALF) and in the in vitro cell model were determined using ELISA kits provided by Invitrogen (USA) for IL-4 (catalog number: 900-TM49), IL-5 (catalog number: 900-M406), IL-13 (catalog number: 900-M207), and IgE (catalog number: EMIGHE) [42].

### MTT assay

The cells under examination were seeded in a 96-well cell culture plate at a density of 3–5 × 10^4^ cells/mL and incubated under normal conditions for 48 h. Subsequently, cells in the hypoxia-related treatment group were exposed to a gas mixture of 0% O_2_, 5% CO_2_, and 95% N2 at a flow rate of 10 L/min for 15 min. The setup was then sealed and placed in a 5% CO_2_, 37 °C incubator for 3 h. MTT solution (10 mg/mL, catalog number: ST316, Beyotime Biotechnology Co., Ltd, Shanghai) was added to the cell suspension, incubated for 4 hours, followed by the addition of dimethyl sulfoxide (DMSO) and shaking for 10 min. The absorbance (OD 490 nm) was measured using a spectrophotometer (Laspec, China) [43].

### EdU experiment

The cells under examination were seeded in a 24-well plate, with cells in the hypoxia-related treatment group exposed to a gas mixture of 0% O_2_, 5% CO_2_, and 95% N_2_ at a flow rate of 10 L/min for 15 min. Subsequently, they were sealed indoors and incubated in a 5% CO_2_, 37 °C incubator for 3 h. Following this, EdU (catalog number: C10310-2, Guangzhou RiboBio Co., LTD, Guangzhou) was added to the culture medium to a concentration of 10 µmol/L and further incubated in the incubator for 2 h. The growth medium was then removed, and the cells were fixed with PBS solution containing 4% paraformaldehyde at room temperature for 15 min. After two washes with PBS containing 3% BSA, the cells were incubated with PBS containing 0.5% Triton-100 at room temperature for 20 min, followed by another two washes with PBS containing 3% BSA. Subsequently, 100 µL of staining solution was added to each well and incubated at room temperature in the dark for 30 min. DAPI staining was carried out for 5 min, the plates were sealed, and 6–10 random fields were observed under a fluorescence microscope (BX63, Olympus, Japan). The number of positive cells in each field was recorded, and the EdU labeling rate was calculated as follows: EdU labeling rate (%) = (Number of positive cells / (Number of positive cells + Number of negative cells)) × 100% [44]. Each experiment is repeated three times.

### Cell cycle analysis

Cell cycle analysis was conducted using flow cytometry, and the cell processing and processing methods were the same as mentioned above. ASMCs cells were fixed overnight in 70% cold ethanol at 4℃, followed by centrifugation at 800 g to remove the supernatant. The cells were then incubated at 37℃ for 30 min in a binding buffer containing 10 µg/mL RNase A. In addition, the cells were stained with 50 µL of propidium iodide (PI, 50 mg/L, catalog number: 40710ES03, YEASEN, Shanghai) for 30 min. Finally, the cell cycle progression was analyzed using flow cytometry (BD Bioscience, BD LSRFortessa, USA) [45].

### Transwell experiments

After collecting and transfecting cells for 48 h, resuspend 10^5^ cells with a serum-free culture medium. Inoculate each well of the upper chamber with 200 µL of cell suspension (2 × 10^4^ cells/well) in the Transwell plate. Add 800 µL of medium with 20% FBS to the lower chamber. After incubation in a 37 ℃ incubator for 24 h, remove the Transwell plate and rinse it twice with PBS. Then, immerse it in formaldehyde for 10 min and rinse it three times with water. Staining was performed using 0.1% crystal violet. After incubation at room temperature for 30 min, samples were rinsed twice with PBS. Cells on the upper surface were gently removed using a cotton ball. The migrated cells were visualized and photographed using an inverted microscope (BX63, Olympus, Japan). Cell migration ability was assessed by counting and analyzing the images with Image J software [46, 47].

### TUNEL staining

Fix the ASMCs from each group with 4% paraformaldehyde at room temperature for 15 min (catalog number: 60536ES60, Yeasen Biotechnology (Shanghai) Co., Ltd., China), and permeabilize them with 0.25% Triton X-100 at room temperature for 20 min. Cultivate cells with 5% bovine serum albumin (BSA, catalog number: 36101ES25, Yeasen Biotechnology (Shanghai) Co., Ltd., China), then stain with the TUNEL (catalog number: C1086, Beyotime Biotechnology Co., Ltd., Shanghai) reagent. Subsequently, the slices were restained with DAPI staining solution (catalog number: C1002, Beyotime Biotechnology Co., Ltd, Shanghai) in the dark. Capture images of apoptotic cells using a confocal microscope (model: LSM 880, Carl Zeiss AG, Germany). TUNEL-positive cells (green fluorescence) indicate apoptotic cells, and DAPI is used to stain the cell nuclei and emits blue fluorescence. The blue fluorescence represents the total number of cells. Five different fields of view were selected for each group to calculate the percentage of apoptotic cells by dividing the number of apoptotic cells by the total number of cells and multiplying by 100% [48].

### Dual luciferase reporter gene assay

Targeted binding of HIF-1α and MDM2 was achieved by using PCR technology to amplify an MDM2 promoter fragment containing the binding region with HIF-1α from the mouse genome in vitro and cloning it into the luciferase reporter vector pGL3-basic plasmid (Catalog number: E1751, Promega, USA). Transfection of 0.4 µg of MDM2 promoter reporter vector alone or co-transfection with oe-NC, oe-HIF-1α vector into ASMCs was carried out using Lipofectamine 3000 (Catalog number: L3000015, Invitrogen, USA) as the transfection reagent. pRL-CMV Renilla luciferase served as the control reporter vector. After transfection for 48 h, cells were collected and lysed. The lysates were centrifuged for 3–5 min, and the supernatant was collected. The relative luciferase activity was analyzed using the Dual-Luciferase Reporter Assay System (Catalog Number: E2261, Promega, USA), with the ratio of firefly luciferase activity to renilla luciferase activity as the measure. Each experiment was repeated 3 times [49, 50].

### Co-immunoprecipitation (Co-IP)

Inoculate ASMCs cells onto a 6 cm plate and treat the cells with 10 µM proteasome inhibitor MG132 (catalog number: HY-13,259, Med Chem Express, Shanghai) for 6 h. Then, collect cells and lyse with NP-40 lysis buffer (Catalog No. P0013F, Beyotime Biotechnology Co., Ltd., Shanghai). Prepare 40 µg total protein as the input group, and prepare the remaining protein at a concentration of 1 mg/mL in a volume of 1 mL and add it to three test tubes. Add P53 antibody (10 µg, catalog number: ab26, Abcam, UK) or IgG antibody (10 µg, catalog number: ab6789, Abcam, UK) to the total protein and then stir slowly overnight at 4 ℃. Add Protein A/G agarose (catalog No: sc-2003, Santa Cruz Biotechnology, USA) and incubate at 4 °C for 4 h. Then, wash three times with pre-chilled TBS solution and analyze the immunoprecipitated proteins by protein immunoblotting analysis [51, 52].

### Analysis of P53 ubiquitination levels

Transfect sh-NC or sh-MDM2 and His-P53 with (or) HA-Ub into ASMCs cells, His-P53 and HA-Ub were constructed and provided by Genechem (Shanghai). After 48 h, incubate with MG132 (10 µM, catalog number: HY-13,259, Med Chem Express, Shanghai) for 8 h, lyse the cells with lysis buffer, and immunoprecipitate the cell lysate with anti-His antibody (catalog number: ab213204, 1:30, Abcam, UK) at 4 °C for 24 h. Then, mix the sample with Protein A-Sepharose beads (Cat. No. 9863, Cell Signaling Technology, USA) and incubate at 4 ℃ for 2 h. Then, wash them with a proteinase buffer and a sample buffer to obtain the target protein. Protein expression levels were detected by immunoblotting analysis (IB) using an anti-ubiquitin antibody (catalog number: 3936, Cell Signaling Technology, USA) [53, 54].

### P53 protein stability measurement

To determine the stability of the P53 protein, ASMCs cells transfected with oe-NC or oe-MDM2 plasmids were treated with 3 µg/L CHX (catalog number: M4879, AbMole, USA) or proteasome inhibitor MG132. After cell lysis using RIPA lysis buffer (catalog number: P0013B, Beyotime Biotechnology Co., Ltd, Shanghai) and centrifugation at 12,000 rpm, protein extraction was performed for immunoblot analysis. Quantification and normalization of P53 levels to GAPDH were performed using ImageJ. Protein extraction was performed at 0 h, 1 h, and 2 h, followed by Western blot analysis to determine the expression level of P53. The degradation curve of P53 was then plotted [55].

### In vivo animal experiments

Mice were randomly divided into six groups, each comprising six mice, as follows: Without asthma group (mice without modeling), Asthma group (mice induced with OVA for asthma), Asthma + Vehicle group (asthmatic mice with blank solution intervention), Asthma + IDF-11,774 group (asthmatic mice intervened with IDF-11,774), Asthma + oe-NC + IDF-11,774 group (asthmatic mice transfected with oe-NC and intervened with IDF-11,774), and Asthma + oe-MDM2 + IDF-11,774 group (asthmatic mice transfected with oe-MDM2 and intervened with IDF-11,774). IDF-11,774 (catalog number: HY-111,387, MedChemExpress, USA) is an inhibitor of HIF-1α. After establishing the asthma model, except for the Control, Model, and Vehicle groups, each group of mice received oral administration of 50 mg/kg IDF-11,774 continuously for 14 days [56, 57].

Mice were intravenously injected via the tail vein with lentivirus (1 × 10^7^ infectious units/mL) for two consecutive doses, each two days apart, starting seven days before the asthma model induction (Day − 7). Subsequently, asthma induction was performed on Day 0. On the 14th day post asthma induction (Day 14), mice were sacrificed for bronchoalveolar lavage fluid (BALF) and lung tissue analysis [22, 58]. .

### Bronchoalveolar lavage fluid (BALF) test

After the experiment ends, we use 75% alcohol to disinfect the skin of the mouse’s neck. Then, use sterilized surgical scissors to make an incision in front of the mouse’s neck to expose the genioglossus muscle and the sides of the sternothyroid muscle. By separating the surrounding tissues, we exposed the trachea. Subsequently, we will insert a 24-gauge indwelling needle into the trachea and secure it with a surgical suture. Connect a sterile syringe with 0.5 mL of phosphate-buffered saline (PBS) containing 1 mM ethylenediaminetetraacetic acid (EDTA) to the indwelling needle and slowly aspirate 5 times. We repeat the above procedure three times to obtain 1.4–1.5 mL of BALF.

Centrifuge BALF at room temperature (500 g, 5 min) to collect the cellular pellet, then resuspend the cells in 100 mL of PBS. Calculate the total number of cells using a hemocytometer. We use Diff-Quik staining (Catalog Number: D030-1-1, Nanjing Jiancheng, China) to observe and count immune cells. We air-dry the cell smears and then fix them with R1 reagent at room temperature. After removing the R1 reagent, we immersed the slices in the R2 reagent for 8 s. After removing the R2 reagent, we stained with the R3 reagent for 8 s. The stained slices are washed with slowly flowing tap water, dehydrated twice with absolute alcohol, and then sealed with neutral resin. Finally, we used an optical microscope (Olympus) to identify and count cell types. Measure the concentration of protein in bronchoalveolar lavage fluid (BALF) using the Bicinchoninic Acid (BCA) Protein Assay Kit (Catalog number: P0009, Beyotime Biotechnology Co., Ltd, Shanghai) [25].

### Lung morphology measurement

Elastica van Gieson staining was used to identify the deposition of collagen fibers and elastic fibers in the lung tissue slices of mice [59, 60]. The procedures were similar to the previously mentioned staining methods. This study simultaneously utilized a 50-line, 100-point grid connected to the microscope eyepiece and employed a point-counting technique to evaluate the bronchial constriction index, smooth muscle area, and epithelial thickness in mice. The bronchial contraction index of mice was evaluated by dividing the number of points where the grid lines intersected with the basement membrane by the square root of the number of points hitting the airway lumen. Each animal underwent 5 measurements. The smooth muscle area and epithelial thickness of the airway were evaluated by dividing the number of points hitting smooth muscle or epithelial cells by the intercepts between grid lines and the basement membrane. Five samples were taken from each animal, and measurements were performed at a magnification of 400 times [61].

### Statistical analysis

Our research used R language, version 4.2.1. The R language is compiled through the integrated development environment RStudio, with version 2022.12.0-353. We use Perl version 5.30.0 for file processing. In addition, we also used GraphPad Prism software, version 8.0. We represent metric data using mean ± standard deviation. We used an independent samples t-test to compare two sets of data [62].

To compare data between different groups, we used one-way analysis of variance (ANOVA). For the comparison of data within the same group at different time points, we used two-way analysis of variance (ANOVA). We performed a post hoc test using Bonferroni. The significance threshold is *P* < 0.05 [63].

## Results

### Identification of HIF-1α as a key gene associated with airway remodeling and inflammation in asthma using multivariate analysis and high-throughput sequencing

Airway remodeling is another major pathological feature of asthma and is equally important in the pathogenesis of asthma as inflammation. Therefore, developing new strategies to reduce airway remodeling and inflammation is crucial for the overall prognosis of asthma patients.

To investigate the key genes influencing airway remodeling and inflammation, we established an asthma mouse model using ovalbumin (OVA) sensitization method. The lung tissues of mice from the Without Asthma group and the Asthma group were subjected to histological examination through tissue slicing. The histological analysis revealed that the lung tissue slices from mice in the Without Asthma group exhibited a normal tissue structure with no signs of inflammation or epithelial damage. In contrast, the lung tissue slices from the Asthma group mice showed extensive alveolar septal thickening, interstitial edema, and infiltration of inflammatory cells. Collagen and elastic fiber deposition on the airway walls were significantly higher in the Asthma group compared to the Without Asthma group, indicating the successful construction of the model (Figure S1A).

**Fig. 1:**
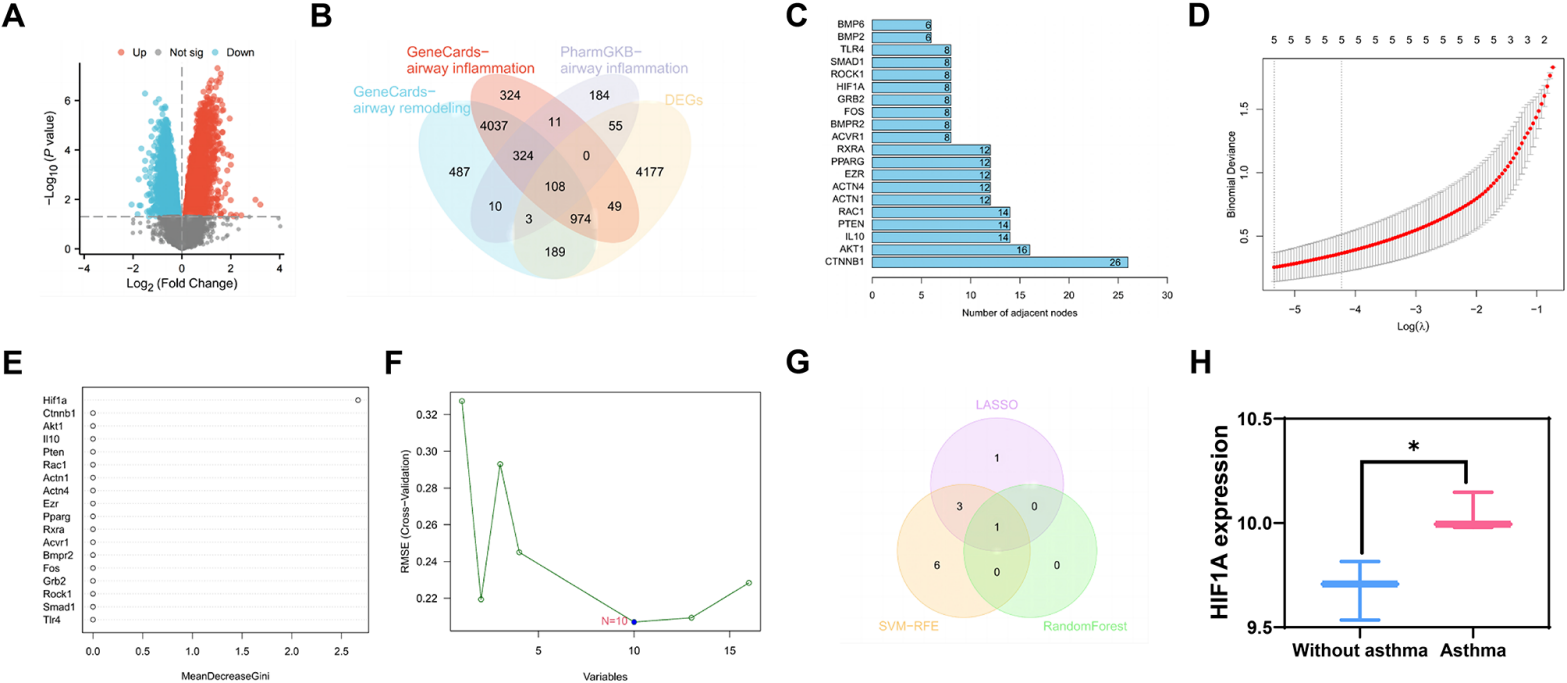
A machine learning algorithm for screening mRNA related to airway remodeling and inflammation. *Note* (**A**) Volcano plot of differentially expressed mRNA between Asthma group and Without asthma group in high-throughput sequencing data of mouse lung tissues; (**B**) Venn diagram showing the intersection of significantly differentially expressed mRNA in Asthma mouse lung tissues in sequencing data, mRNA related to airway remodeling and inflammation in the Genecards database, and mRNA related to airway inflammation in the PharmGKB database; (**C**) Statistics of the number of adjacent nodes of core genes in the gene interaction network graph, where the x-axis represents the number of adjacent nodes and the y-axis represents gene names; (**D**) LASSO regression coefficient selection plot; (**E**) Random forest algorithm result plot; (**F**) SVM-RFE analysis result plot; (**G**) Venn diagram showing the intersection of Asthma-related mRNA selected by three machine learning algorithms: LASSO regression, random forest algorithm, and SVM-RFE; (**H**) Expression of HIF-1α in the sequencing data, displaying the results as the logarithmic values of gene expression (with 3 cases without asthma and 3 cases with asthma). In the volcano plot, blue dots represent significantly downregulated mRNA in the Asthma group, red dots represent significantly upregulated mRNA in the Asthma group, and gray dots represent mRNA with no significant difference. *Note* * indicates *P* < 0.05 compared to the Without asthma group

Hence, we conducted high-throughput sequencing on the lung tissues of mice from the Without Asthma group and the Asthma group, followed by data processing and differential analysis. The results revealed 5555 significantly different mRNAs in the lung tissues of mice in the Asthma group compared to those in the Without Asthma group (Fig. 1A).

Six thousand one hundred thirty-two genes associated with airway remodeling and 5827 genes associated with airway inflammation were obtained from the Genecards database. Additionally, 695 genes associated with airway inflammation were obtained from the PharmGKB database. By performing an intersection analysis between differentially expressed genes (DEGs) and genes obtained from databases, we identified 108 candidate target genes (Fig. 1B).

Further interaction analysis was conducted for these 108 candidate target genes, and a gene interaction network was constructed (Figure S1B). The number of adjacent nodes for each gene in the network graph was calculated, and it was found that there were 18 genes with 8 or more adjacent nodes. These genes are CTNNB1, AKT1, IL10, PTEN, RAC1, ACTN1, ACTN4, EZR, PPARG, RXRA, FOS, ACVR1, BMPR2, GRB2, HIF1A, ROCK1, SMAD1, TLR4 (Fig. 1C).

Among them, RAC1, TLR4, CTNNB1, SMAD1, EZR, and RXRA were downregulated in the sequencing data, while ACVR1, PPARG, ACTN1, HIF1A, PTEN, ACTN4, GRB2, BMPR2, FOS, and ROCK1 were upregulated in the sequencing data (Figure S1C). Enrichment analysis of these 18 genes revealed an association between these genes and various lung diseases, including obstructive lung disease, interstitial lung disease, chronic obstructive pulmonary disease, bronchial disease, and asthma (Figure S1D).

Next, we conducted a multivariate Cox analysis using LASSO regression on these 18 genes (Fig. 1D). We also assessed the importance of the genes using the random forest algorithm (Fig. 1E) and extracted asthma-related genes using the SVM-RFE analysis method (Fig. 1F). Ultimately, we identified a key mRNA: HIF-1α (Fig. 1G), whose expression profile in the sequencing data is shown in Fig. 1H.

Based on the above results, we have identified a gene, HIF-1α, which is associated with airway remodeling and inflammation caused by asthma, and it is expressed at higher levels in our asthma model.

### HIF-1α enhances viability, proliferation, and migration of asmcs in hypoxic conditions while suppressing apoptosis

To investigate the role of HIF-1α in the ASMCs hypoxic model, ASMCs were isolated from the bronchi of Asthma mice, and their cellular quality was assessed. Immunofluorescence staining was conducted to detect the expression of α-SMA, which revealed a significant expression of α-SMA. The isolated cells demonstrated a purity of over 95%, indicating a high-quality isolation of ASMCs suitable for subsequent analysis (Figure S2A). Then, the silencing effect of sh-HIF-1α in ASMCs was confirmed by RT-qPCR. The results showed that the silencing effect of sh-HIF-1α-1 was the best. Therefore, sh-HIF-1α-1 was selected for the subsequent experiments (Figure S2B).

**Fig. 2:**
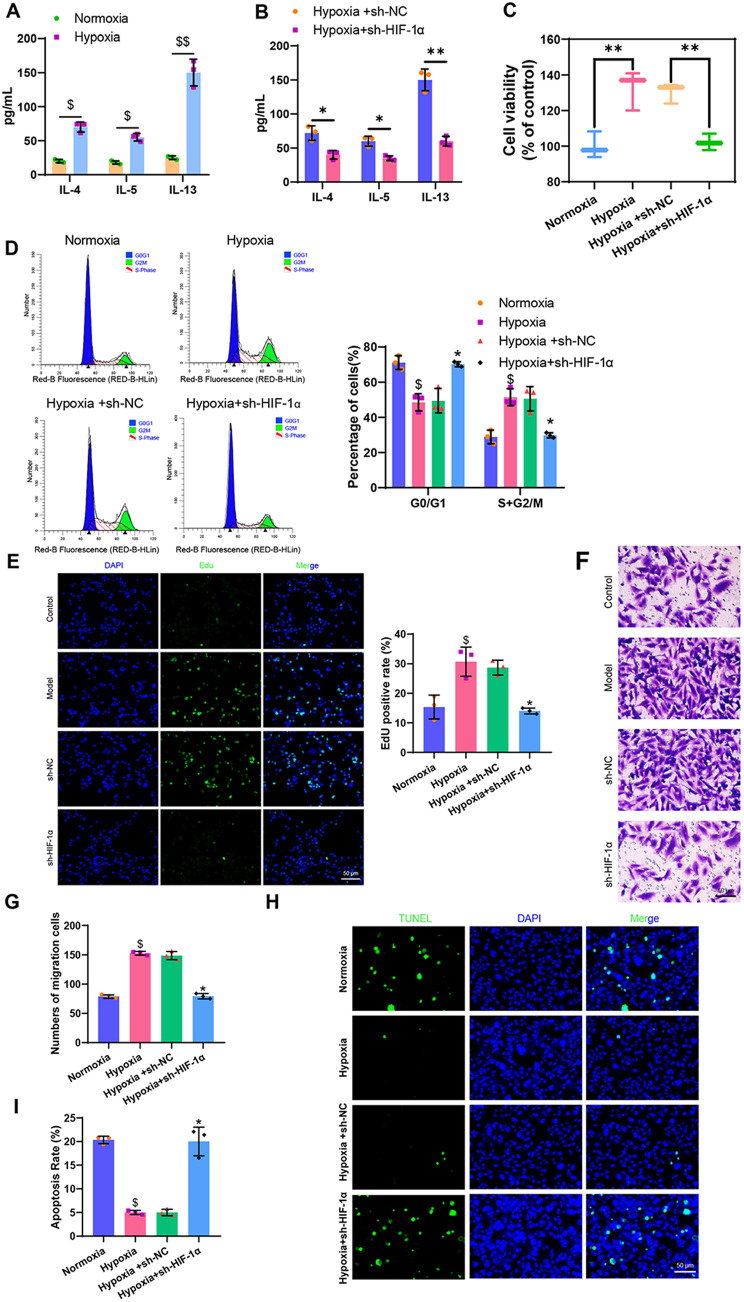
Effects of HIF-1α on ASMCs’ biological functions. *Note* (**A**) ELISA detection of inflammation factor levels in ASMCs before and after modeling; (**B**) ELISA detection of inflammation factor levels in cells from each group; (**C**) MTT assay to measure cell viability in each group, “%control” refers to the cell viability of each group compared to the Normoxia group; (**D**) Flow cytometry analysis of cell cycle changes in each group; (**E**) EdU experiment to evaluate cell proliferation capacity in each group (scale bar: 20 μm); (**F**) Transwell assay to assess cell migration ability in each group; (**G**) Quantification of Transwell assay results; (**H**) TUNEL assay to measure cell apoptosis rate in each group (scale bar = 50 μm); (**I**) Quantification of TUNEL assay results. Statistical significance was denoted as follows: * for *P* < 0.05 compared to the Hypoxia + sh-NC group, ** for *P* < 0.01 compared to the Hypoxia + sh-NC group, $ for *P* < 0.05 compared to the Normoxia group, and $$ for *P* < 0.01 compared to the Normoxia group. Each cell experiment was replicated three times

Subsequently, ASMCs isolated were cultured under hypoxic conditions while concurrently knocking down HIF-1α. RT-qPCR was performed to measure the expression levels of HIF-1α before and after model construction, and ELISA was utilized to determine the levels of the inflammatory cytokines IL-4, IL-5, and IL-13 in the model cells. The results indicated a significant increase in the levels of HIF-1α, IL-4, IL-5, and IL-13 in ASMCs under hypoxia compared to the normoxia group, indicating the successful establishment of a hypoxia-induced in vitro cellular model. Furthermore, compared to the Hypoxia + sh-NC group, the levels of IL-4, IL-5, and IL-13 decreased significantly in the Hypoxia + sh-HIF-1α group (Fig. 2A-B, Figure S2C).

To further investigate the biological effects of HIF-1α on ASMCs cellular model, we conducted MTT assays, flow cytometry, EdU assay, Transwell assay, and TUNEL staining. The results revealed that compared to the normoxia group, the cells in the hypoxia group exhibited significantly increased viability, proliferation, and migratory capacity, with a notable increase in the S + G2/M phase cell proportion and a significant decrease in apoptosis levels. In contrast, when compared to the Hypoxia + sh-NC group, cells in the Hypoxia + sh-HIF-1α group showed significantly reduced viability, proliferation, and migratory capacity, along with a decrease in the S + G2/M phase cell proportion and a significant increase in apoptosis levels (Fig. 2C-I).

In summary, HIF-1α could promote cell viability, proliferation, migration, and inflammatory response in ASMCs under hypoxic conditions and inhibit cell apoptosis.

### HIF-1α modulation influences P53 protein expression in ASMCs under hypoxic conditions

To identify the downstream target genes of HIF-1α, we performed GO, KEGG, and PANTHER pathway enrichment analysis on HIF-1α. The GO analysis results indicate that the target gene may be involved in the following biological processes: regulation of RNA polymerase II promoter transcription in response to hypoxia/oxidative stress and gas homeostasis, etc. The cellular environment may include RNA polymerase II transcription regulatory complexes, neuronal dendritic cytoplasm, and axonal cytoplasm. Possible molecular functions include P53 binding, nuclear receptor binding, and macroprotein transcriptional co-regulator binding. The KEGG and PANTHER analysis results show that the target genes may participate in regulatory pathways such as the hypoxia-induced HIF activation response and the HIF-1 signaling pathway (Figure S3). The above analysis results indicate that HIF-1α is closely related to cellular hypoxia and may regulate P53 protein expression.

**Fig. 3:**
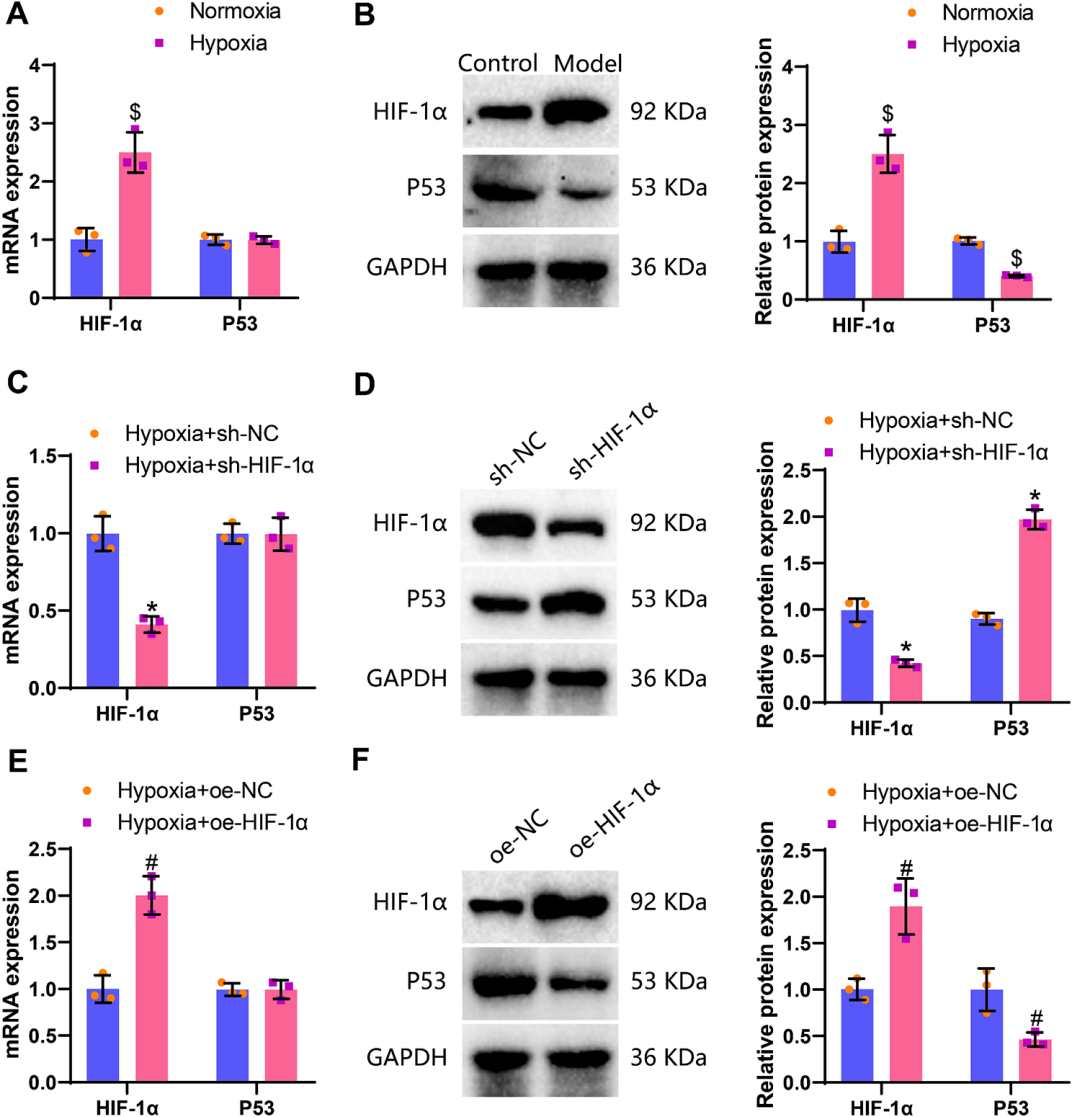
Effects of hypoxia on the expression levels of HIF-1α and P53. *Note* (**A**) RT-qPCR detection of HIF-1α and P53 levels in ASMCs of the Normoxia group and Hypoxia group; (**B**) Western blot detection of HIF-1α and P53 protein expression levels in ASMCs of the Normoxia group and Hypoxia group; (**C**) RT-qPCR detection of HIF-1α and P53 levels in ASMCs of the Hypoxia + sh-NC group and Hypoxia + sh-HIF-1α group; (**D**) Western blot detection of HIF-1α and P53 protein expression levels in ASMCs of the Hypoxia + sh-NC group and Hypoxia + sh-HIF-1α group; (**E**) RT-qPCR detection of HIF-1α and P53 levels in ASMCs of the Hypoxia + oe-NC group and Hypoxia + oe-HIF-1α group; (**F**) Western blot detection of HIF-1α and P53 protein expression levels in ASMCs of the Hypoxia + oe-NC group and Hypoxia + oe-HIF-1α group. * denotes *P* < 0.05 compared to the Hypoxia + sh-NC group, # denotes *P* < 0.05 compared to the Hypoxia + oe-NC group, $ denotes *P* < 0.05 compared to the Normoxia group; each cell experiment was replicated three times

To validate the aforementioned analyses, we employed a lentivirus transduction method to either overexpress or knock down HIF-1α, and utilized RT-qPCR and Western blot techniques to assess the expression levels of P53 in an in vitro cellular model of ASMCs. The experimental findings revealed that, compared to the Normoxia group, the mRNA and protein expression of HIF-1α significantly increased in the Hypoxia group, while there was no significant change in the mRNA level of the P53 gene, but a notable decrease in protein expression was observed. In comparison to the Hypoxia + sh-NC group, the Hypoxia + sh-HIF-1α group displayed no significant alteration in the mRNA level of the P53 gene, however, a significant increase in protein expression was noted. Furthermore, when compared to the Hypoxia + oe-NC group, the Hypoxia + oe-HIF-1α group exhibited no significant change in the mRNA level of the P53 gene, yet a significant decrease in protein expression levels was observed (Fig. 3A-F).

The above results suggest that hypoxia induction upregulates HIF-1α and inhibits the expression of P53 protein in ASMCs.

### HIF-1α promotes P53 ubiquitination in ASMCs via MDM2 regulation under hypoxic conditions

In the aforementioned experiment, it was demonstrated that the upregulation of HIF-1α could inhibit the expression of the P53 protein, but the mechanism by which HIF-1α negatively regulates P53 protein expression has not yet been elucidated. The protein levels of P53 are primarily regulated by post-translational modifications such as ubiquitination, phosphorylation, and acetylation, where ubiquitination can regulate its biological function through promoting P53 degradation [64–66]. Additionally, it has been shown in the literature that HIF-1α can interact with ubiquitin-related modifier proteins to promote the development of asthma [67]. Therefore, it is hypothesized that HIF-1α may regulate the ubiquitin ligase, promoting the ubiquitination and degradation of the P53 protein, thereby inhibiting the expression of P53.

**Fig. 4:**
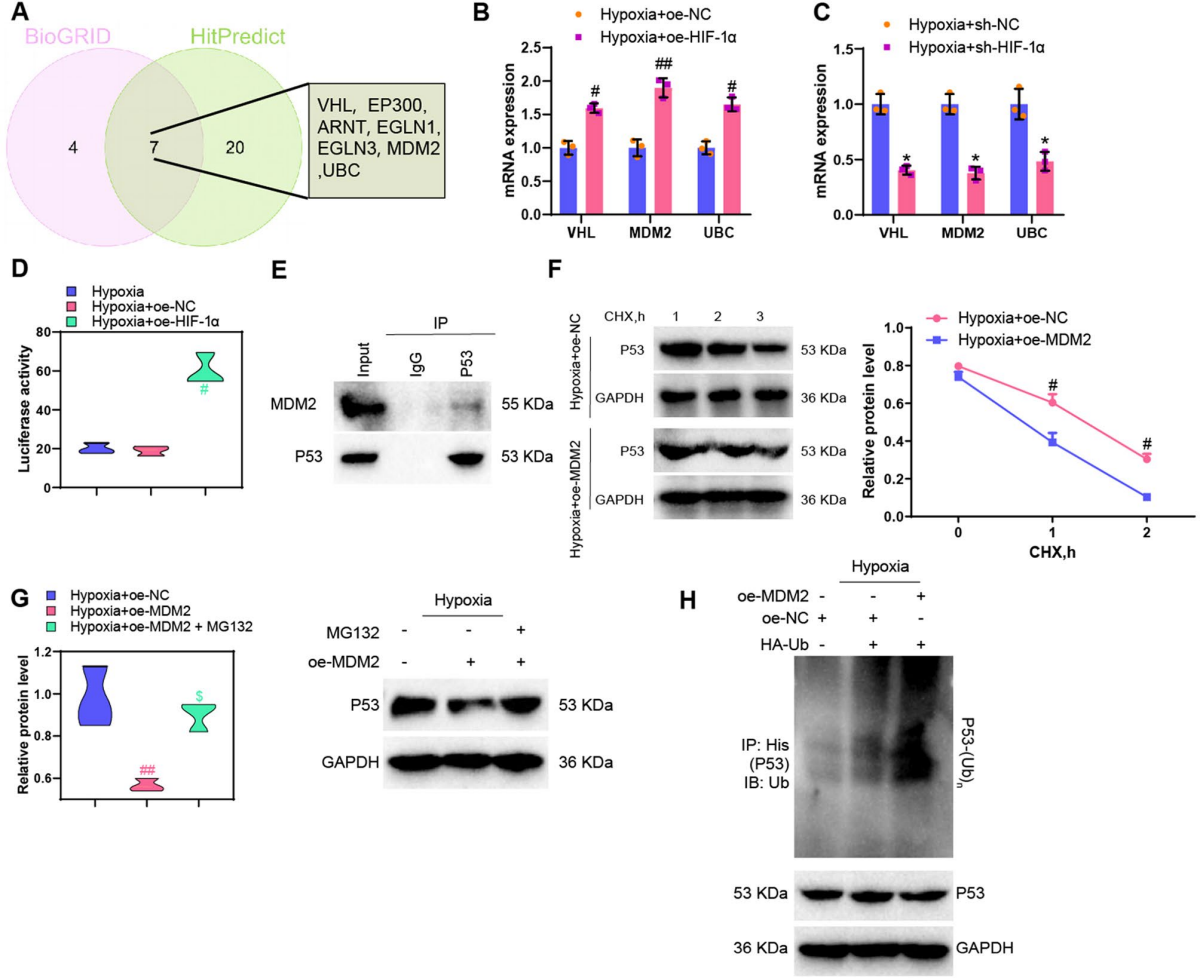
Regulation of MDM2 and P53 protein by HIF-1α. *Note* (**A**) Venn diagram showing the intersection of genes predicted to bind to HIF-1α in the BioGRID and HitPredict databases; (**B**)-(**C**) RT-qPCR detection of VHL, MDM2, and UBC expression in ASMCs cell models with HIF-1α overexpression or knockdown; (**D**) Dual-luciferase reporter gene experiment for detecting the binding relationship between HIF-1α and the MDM2 promoter in ASMCs cell models; (**E**) Co-IP experiment to detect the endogenous binding of MDM2 with P53 in ASMCs cell models; (**F**) Protein blotting to examine the P53 protein levels in ASMCs cells treated with protein synthesis inhibitor CHX; (**G**) Protein blotting to evaluate P53 protein expression in ASMCs cell models after treatment with proteasome inhibitor MG132; (**H**) Analysis of ubiquitination levels of P53 in ASMCs cells. * indicates *P* < 0.05 compared to the Hypoxia + sh-NC group, ** indicates *P* < 0.01 compared to the Hypoxia + sh-NC group, # indicates *P* < 0.05 compared to the Hypoxia + oe-NC group, ## indicates *P* < 0.01 compared to the Hypoxia + oe-NC group

To verify the above conjecture, we used the BioGRID database to predict the binding protein genes of HIF-1α and obtained 437 binding protein genes. There were 11 genes with Evidence greater than 10. The prediction results obtained from the HitPredict database show that 426 protein genes bind to HIF-1α. Out of these, 27 genes have an Interaction Score greater than 0.7. After taking the intersection of the two sets, 7 common genes are obtained, of which only 3 are ubiquitin ligase genes (VHL, MDM2, and UBC) (Fig. 4A).

In our study, we utilized RT-qPCR experiments to assess the expression levels of VHL, MDM2, and UBC in an in vitro hypoxic cell model with knocked down or overexpressed HIF-1α. The results indicated that in ASMCs where HIF-1α was simultaneously knocked down or overexpressed under hypoxic conditions, MDM2 (FC = 2.0 and FC = 2.2) exhibited the most significant differences compared to VHL (FC = 1.7 and FC = 1.75) or UBC (FC = 1.8 and FC = 1.55). Therefore, we identified MDM2 as the most likely ubiquitin ligase to bind with HIF-1α (Fig. 4B-C). The literature has reported that the E3 ubiquitin ligase MDM2 could bind to the P53 protein and promote its ubiquitination and degradation [65].

Furthermore, we validated the direct regulatory relationship between HIF-1α and MDM2 through a dual-luciferase reporter assay. Co-IP experiment was conducted to determine the endogenous binding relationship between MDM2 and P53. The results showed that the overexpression of HIF-1α enhanced the activity of MDM2 promoter. Co-IP experiment confirmed the interaction between MDM2 and P53 (Fig. 4D-E).

Subsequently, ASMCs were transfected with oe-MDM2 to conduct in vitro ubiquitination assays and protein stability experiments to delineate the role of MDM2 on P53. The results of the protein stability experiments revealed that following treatment with the protein synthesis inhibitor cycloheximide (CHX), the protein level of P53 decreased more rapidly in the Hypoxia + oe-MDM2 group compared to the Hypoxia + oe-NC group (Fig. 4F), indicating that overexpression of MDM2 reduces the protein stability of P53 and increases its degradation. Then, we treated ASMCs cells with the proteasome inhibitor MG132 (10 µM) and observed an increase in the stability of P53 protein (Fig. 4G). The in vitro ubiquitination assay results of P53 indicate that overexpression of MDM2 increases the ubiquitination level of P53, suggesting that MDM2 mediates the ubiquitination of P53 (Fig. 4H). The above experimental results all demonstrate that MDM2 could promote the degradation of P53 by regulating ubiquitination.

Subsequently, MDM2 was knocked down in ASMCs, and the RT-qPCR experiment validated the silencing effect of sh-MDM2 in ASMCs. The results showed that sh-MDM2-3 had the best silencing effect. Therefore, sh-MDM2-3 was chosen for subsequent experiments (Figure S4A). The RT-qPCR results revealed that, compared to the Hypoxia + oe-NC group, the expression levels of HIF-1α and MDM2 significantly increased in ASMCs cells of the Hypoxia + oe-HIF-1α group, while there was no significant change in the expression level of P53. In the Hypoxia + oe-MDM2 group, the expression level of MDM2 significantly increased in the ASMCs cells, with no significant change observed in the expression levels of HIF-1α and P53 (Figure S4B-C). When juxtaposed with the Hypoxia + sh-NC group, the expression levels of HIF-1α and MDM2 notably decreased in ASMCs cells of the Hypoxia + sh-HIF-1α group, whereas there was no significant change in the expression level of P53. Additionally, in comparison to the Hypoxia + sh-NC group, the expression level of MDM2 significantly decreased in the Hypoxia + sh-MDM2 group, while no significant changes were observed in the expression levels of HIF-1α and P53 (Figure S4D-E).

The above results indicate that hypoxia-induced HIF-1α promotes the ubiquitination of P53 protein in ASMCs by regulating MDM2.

### HIF-1α mediates ASMCs’ biological functions under hypoxia through the MDM2/P53 signaling axis

**Fig. 5:**
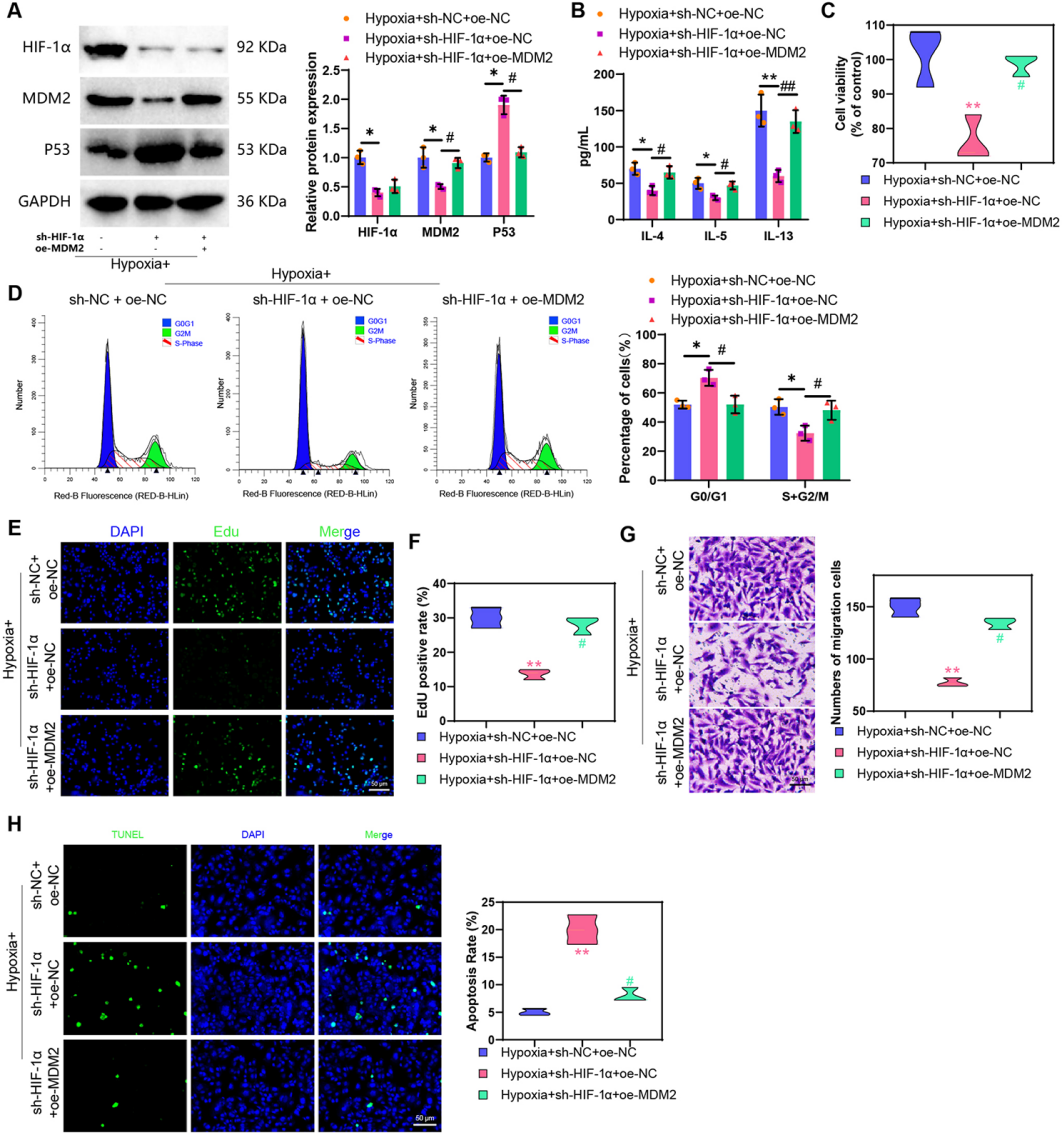
Effects of the HIF-1α/MDM2/P53 axis on ASMCs’ biological functions. *Note* (**A**) Western blot detection of HIF-1α, MDM2, and P53 protein expression levels in cells from each group; (**B**) ELISA detection of inflammation factor levels in cells from each group; (**C**) MTT assay to measure cell viability in each group, “%control” refers to the cell viability of each group compared to the Normoxia group; (**D**) Flow cytometry analysis of cell cycle changes in each group; (**E**) EdU experiment to evaluate cell proliferation capacity in each group (scale bar: 20 μm); (**F**) Quantification of EdU experiment results; (**G**) Transwell assay to assess cell migration ability in each group; (**H**) TUNEL assay to measure cell apoptosis rate in each group (scale bar = 50 μm). Statistical significance was indicated by (*) when comparing with the Hypoxia + sh-NC + oe-NC group where *P* < 0.05, (**) when compared with the Hypoxia + sh-NC + oe-NC group where *P* < 0.01, (#) when compared with the Hypoxia + sh-HIF-1α + oe-NC group where *P* < 0.05, and (##) when compared with the Hypoxia + sh-HIF-1α + oe-NC group where *P* < 0.01. The cell experiments were repeated three times for reliability

To further investigate whether low oxygen-induced HIF-1α regulates the biological functions of ASMCs’ under hypoxic conditions by modulating the MDM2/P53 signaling axis, we utilized lentiviral knockdown of HIF-1α and overexpression of MDM2. Through Western blot analysis, we observed that in ASMCs under hypoxia, the expression of HIF-1α and MDM2 significantly decreased while P53 protein expression increased in the Hypoxia + sh-HIF-1α + oe-NC group compared to the Hypoxia + sh-NC + oe-NC group. Moreover, in the Hypoxia + sh-HIF-1α + oe-MDM2 group, P53 protein expression significantly decreased, MDM2 protein expression increased, and HIF-1α protein expression remained unchanged compared to the Hypoxia + sh-HIF-1α + oe-NC group (Fig. 5A).

Subsequently, we quantified the levels of inflammatory cytokines IL-4, IL-5, and IL-13 in model cells through ELISA experiments. The results revealed that compared to the Hypoxia + sh-NC + oe-NC group, the levels of IL-4, IL-5, and IL-13 significantly decreased in ASMCs of the Hypoxia + sh-HIF-1α + oe-NC group. Moreover, in ASMCs of the Hypoxia + sh-HIF-1α + oe-MDM2 group, the levels of IL-4, IL-5, and IL-13 significantly increased when compared to the Hypoxia + sh-HIF-1α + oe-NC group (Fig. 5B).

In addition, we assessed cell viability using the MTT assay, cell division and proliferation capabilities through flow cytometry and EdU experiments, cell migration ability via Transwell assay, and apoptosis status through TUNEL staining. The results demonstrated that compared to the Hypoxia + sh-NC + oe-NC group, the cell viability, proliferation, and migration abilities significantly decreased in the Hypoxia + sh-HIF-1α + oe-NC group. Furthermore, there was a significant decrease in the proportion of cells in the S + G2/M phase and a notable increase in apoptosis levels. In contrast, when comparing the Hypoxia + sh-HIF-1α + oe-NC group to the Hypoxia + sh-HIF-1α + oe-MDM2 group, the cell viability, proliferation, and migration abilities significantly increased. Additionally, there was a significant increase in the proportion of cells in the S + G2/M phase and a notable decrease in apoptosis levels in the Hypoxia + sh-HIF-1α + oe-MDM2 group (Fig. 5C-H).

The above results indicate that HIF-1α promotes the cell viability, proliferation, migration, and inflammation response of ASMCs in a hypoxic model by regulating the MDM2/P53 axis while inhibiting cell apoptosis.

### HIF-1α amplifies airway inflammation and remodeling via MDM2/P53 Axis in asthmatic mice; IDF-11,774 mitigates these effects

Through in vitro cell experiments, it has been proven that HIF-1α could promote cell viability, proliferation, migration, and inflammatory response in the low oxygen model of ASMCs by regulating the MDM2/P53 axis while inhibiting cell apoptosis. To verify whether this mechanism affects airway inflammation and remodeling in asthmatic mice, we constructed an asthmatic mouse model using the OVA sensitization method and knocked down HIF-1α while overexpressing MDM2 through intravenous injection of a lentivirus.

**Fig. 6:**
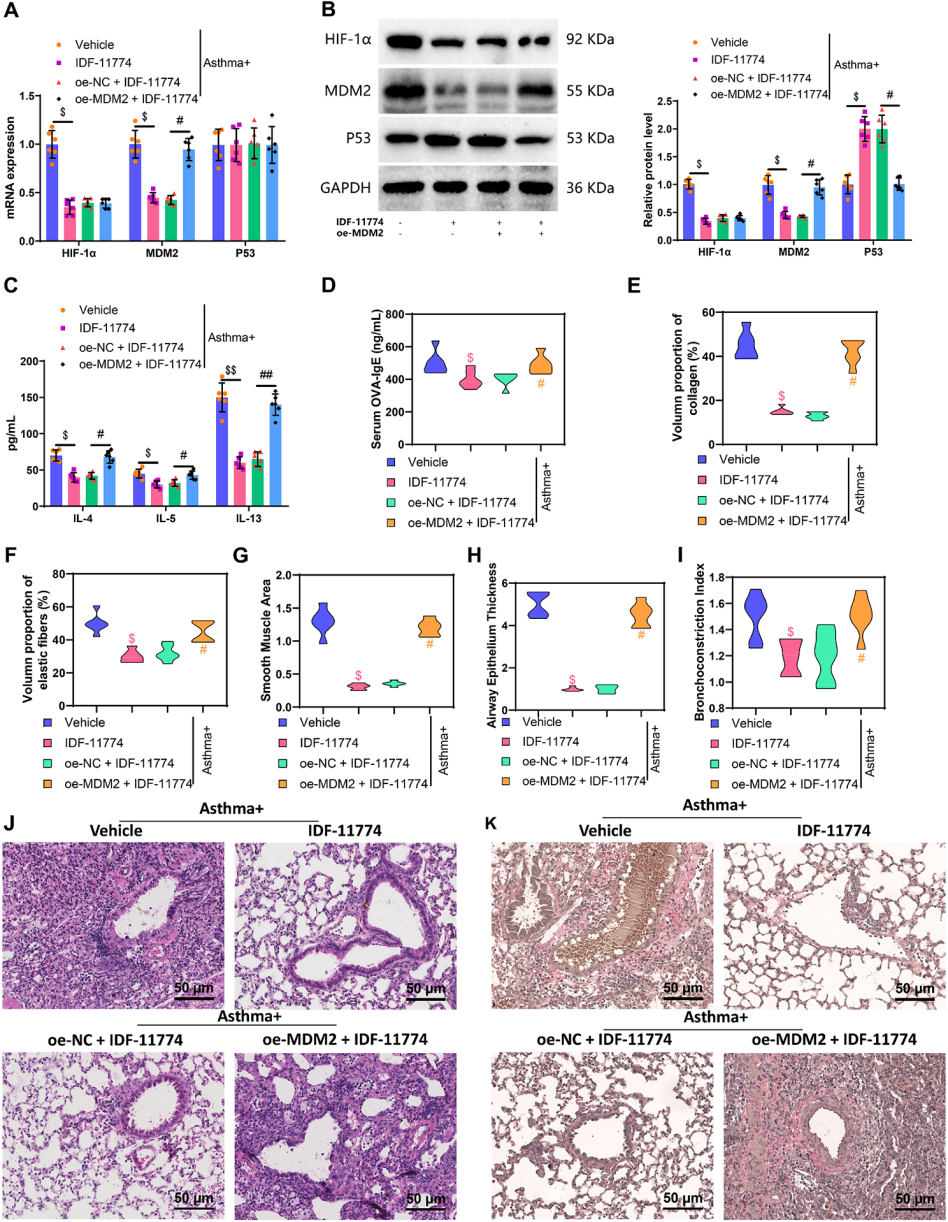
Effects of HIF-1α-regulated MDM2/P53 signaling axis on airway inflammation and remodeling in mice. *Note* (**A**) RT-qPCR detection of HIF-1α, MDM2, and P53 expression levels in mouse lung tissues from each group; (**B**) Western blot detection of HIF-1α, MDM2, and P53 protein expression levels in mouse lung tissues from each group; (**C**)-(**D**) ELISA detection of inflammation factors in bronchoalveolar lavage fluid (BALF) and IgE levels in serum from each group; (**E**) Statistical analysis of collagen protein volume ratio in airway walls; (**F**) Statistical analysis of elastic fiber volume ratio in airway walls; (**G**) Statistical analysis of airway smooth muscle area; (**H**) Statistical analysis of airway epithelial thickness; (I) Statistical analysis of bronchoconstriction index; (**J**) H&E evaluation of lung tissue pathology in each group (scale bar: 50 μm); (**K**) Airway pathological changes in the groups of mice were assessed using Elastica van Gieson (scale: 50 μm), with red representing collagen protein and elastic fiber in the bronchial wall. The symbols # and ## denoted significance when comparing with the Asthma + oe-NC + IDF-11,774 group, where *P* < 0.05 and *P* < 0.01, respectively. Similarly, $ and $$ indicated significance when comparing with the Asthma + Vehicle group, where *P* < 0.05 and *P* < 0.01, respectively. Each group included six mice for the analysis

Initially, our RT-qPCR and Western blot analyses revealed that compared to the mice in the “Without asthma” group, those in the “Asthma” group exhibited significantly increased mRNA and protein expression levels of HIF-1α and MDM2, along with a notable decrease in P53 protein expression. Notably, there were no significant changes observed in the mRNA expression levels (Figure S5A-B). Previous studies have indicated that IDF-11,774 acts as an inhibitor of HIF-1α, suppressing its expression [68, 69]. In sh-HIF-1α ASMC cells, it was confirmed that after HIF-1α was knocked down, IDF-11,774 could not significantly reduce the expression of HIF-1α and MDM2, which confirmed that the biological effects of IDF-11,774 are exerted by targeting HIF-1α (Figure S5C). In comparison to the Asthma + Vehicle group, mice in the Asthma + IDF-11,774 group exhibited a significant decrease in the expression levels of HIF-1α and MDM2 in lung tissues, accompanied by a significant increase in P53 protein expression, while mRNA expression levels remained unchanged. When compared to the Asthma + oe-NC + IDF-11,774 group, the Asthma + oe-MDM2 + IDF-11,774 group showed no significant difference in HIF-1α expression levels in lung tissues. However, MDM2 expression significantly increased, P53 protein expression significantly decreased, and mRNA expression levels remained unchanged (Fig. 6A-B). Bronchoalveolar lavage fluid (BALF) and mouse serum were collected from each group of mice, and the levels of cell inflammatory factors in BALF and IgE in serum were measured through ELISA. The results revealed a significant increase in IgE levels in the serum of mice in the Asthma group compared to the “Without asthma” group. Additionally, compared to the Asthma + Vehicle group, the IDF-11,774 group exhibited a significant decrease in IL-4, IL-5, IL-13, and IgE. Furthermore, compared to the Asthma + oe-NC + IDF-11,774 group, the Asthma + oe-MDM2 + IDF-11,774 group demonstrated a significant increase in IL-4, IL-5, IL-13, and IgE levels (Fig. 6C-D, Figure S5D).

Measurement of the volume ratio of collagen and elastic fibers in the airway walls, as well as the area of airway smooth muscle, epithelial thickness, and bronchoconstriction index, alongside histopathological examination of the mouse lungs using H&E staining, were conducted to observe pathological changes. The results demonstrated that in comparison to the “Without asthma” group, mice in the Asthma group exhibited a significant increase in the volume ratio of collagen and elastic fibers in the airway walls, along with a notable increase in the area of airway smooth muscle, epithelial thickness, and bronchoconstriction index (Figure S5E-I). In the Asthma + Vehicle group, alveolar septa ruptured, leading to substantial infiltration of inflammatory cells into the alveolar septa and spaces. Conversely, compared to the Asthma + Vehicle group, the Asthma + IDF-11,774 group showed restored alveolar septa, significant reduction in inflammatory cell infiltration in alveolar septa and spaces, significant decrease in the deposition of collagen and elastic fibers in the airway walls, and notable reductions in the areas of airway smooth muscle, epithelial thickness, and bronchoconstriction index. Moreover, in contrast to the Asthma + oe-NC + IDF-11,774 group, the Asthma + oe-MDM2 + IDF-11,774 group exhibited a significant increase in inflammatory cell infiltration in alveolar septa and spaces, substantial deposition of collagen and elastic fibers in the airway walls, and marked increases in the areas of airway smooth muscle, epithelial thickness, and bronchoconstriction index (Fig. 6E-K).

The results indicate that HIF-1α exacerbates airway inflammation and induces airway remodeling by regulating the MDM2/P53 axis, while IDF-11,774 could reverse this effect.

## Discussion

In this study, we observed upregulation of HIF-1α in a mouse model of asthma, consistent with previous research [70]. However, we further discovered that HIF-1α is upregulated in the hypoxic airway smooth muscle cells (ASMCs) model. This discovery highlights the complex role of HIF-1α in airway remodeling and inflammation. Previous studies mainly focused on the global effects of HIF-1α, while this study delves into the cellular level and reveals its crucial regulatory role in ASMCs [71].

This study initially revealed genes related to asthma-associated airway remodeling and inflammation through high-throughput sequencing and bioinformatics analysis [3]. Although airway remodeling has been extensively studied, our research offers more in-depth insights at the molecular and genetic levels [72]. This comprehensive analytical approach not only deepens our understanding of airway remodeling but also provides more precise targets for future therapies.

Mechanistically, this study further explored how hypoxic conditions induce the upregulation of HIF-1α, and exacerbate airway inflammation and remodeling through the MDM2/P53 axis. Through in vivo and in vitro experiments, we revealed the mechanism by which hypoxia-induced HIF-1α promotes P53 ubiquitination via MDM2, thus affecting the viability, division, proliferation, migration, and inflammatory responses of ASMCs [19]. Although previous studies have highlighted the importance of the MDM2/P53 axis in many biological processes, its role in airway remodeling and asthma pathology has not been thoroughly explored [73–75]. Compared to previous research, we investigated more deeply the role of hypoxic environments in the pathogenesis of asthma, especially in terms of airway remodeling [76]. This discovery provides a new perspective for understanding the complex pathophysiology of asthma. Our study also found for the first time that the compound IDF-11,774 can reverse the effects of HIF-1α. Studies have shown that IDF-11,774 can target HSP70 and indirectly reduce HIF-1α expression [69], suggesting that IDF-11,774 might have non-target effects besides directly targeting the suppression of HIF-1α expression. Previous research has not covered the potential therapeutic role of this compound in asthma. This finding opens a new direction for asthma drug therapy and aids in future clinical applications.

**Fig. 7:**
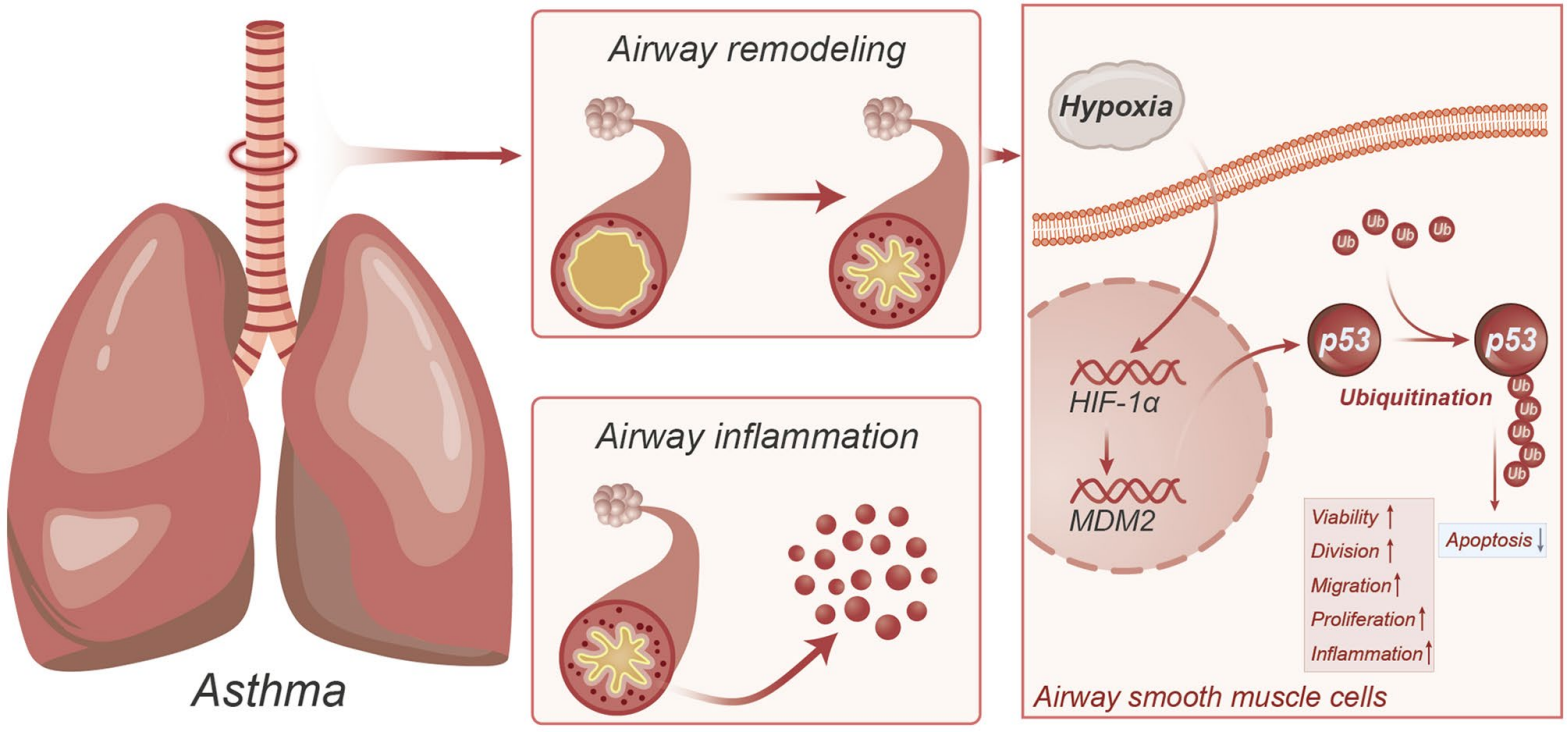
Molecular mechanism diagram illustrating the upregulation of HIF-1α induced by hypoxia, promoting P53 ubiquitination through the regulation of MDM2, exacerbating airway inflammation and inducing airway remodeling

In summary, we can preliminarily conclude the following: Asthma-induced hypoxia responses can upregulate HIF-1α, which, through regulating MDM2, promotes P53 ubiquitination, further enhancing the viability, proliferation, migration, and inflammatory responses of ASMCs and inhibiting apoptosis, thereby exacerbating asthma-induced airway inflammation and inducing airway remodeling (Fig. 7).

The discovery of HIF-1α, MDM2, and P53 provides potential therapeutic targets. By studying the process of hypoxia-induced upregulation of HIF-1α and regulation of MDM2 to promote P53 ubiquitination, this research helps deepen the understanding of the molecular mechanisms of airway remodeling and inflammation. These mechanisms play a key role in respiratory diseases such as bronchial asthma [77]. Targeting these molecules could lead to the development of more effective drugs to treat airway inflammation and bronchial asthma. This research could also aid in the early diagnosis of bronchial asthma and the prediction of disease progression. The aberrant expression of these molecules might serve as biomarkers, guiding personalized treatment plans. Additionally, these findings may also provide insights and applications for other diseases related to hypoxia and inflammatory responses, such as cardiovascular diseases and cancer.

This study used a mouse model for experimentation, and while they simulate human diseases in many aspects, they may not fully match human asthma in certain aspects, limiting the generalization of the research results. The human body’s biological processes are extremely complex, and the interaction between HIF-1α, MDM2, and P53 may only be a part of the whole picture. Research may have overlooked other important factors and pathways. The treatment for these molecules may have unforeseen side effects. Any new treatment method must undergo rigorous clinical trials to validate its safety and effectiveness [78].

Future research needs to conduct clinical trials in a broader population to validate whether these molecules could serve as effective diagnostic markers or therapeutic targets. Further interdisciplinary collaboration may promote understanding of the roles of these molecules in other diseases and accelerate drug development and clinical applications. Through the improvement and innovation of new technologies, such as more precise gene editing and imaging techniques, deeper insights and more precise intervention methods could be provided [79].

This study has important scientific and clinical value for understanding the complex mechanisms of airway inflammation and remodeling, providing new directions and possibilities for future diagnosis and treatment. However, further research is needed to overcome existing limitations and fully leverage the potential opportunities in this field. Therefore, we will continue to explore this direction in the future.

## Electronic supplementary material

Below is the link to the electronic supplementary material.

Supplementary Material 1

Supplementary Material 2

Supplementary Material 3

Supplementary Material 4

Supplementary Material 5

Supplementary Material 6

Supplementary Material 7

## Data Availability

The data that supports the findings of this study are available on request from the corresponding author upon reasonable request.
