# Supplementary figures and images for: Novel hypoxia-induced HIF-1αactivation in asthma pathogenesis
Source: Respir Res. 2024 Jul 25;25:287. doi: 10.1186/s12931-024-02869-0 (PMC11282634; doi:10.1186/s12931-024-02869-0)

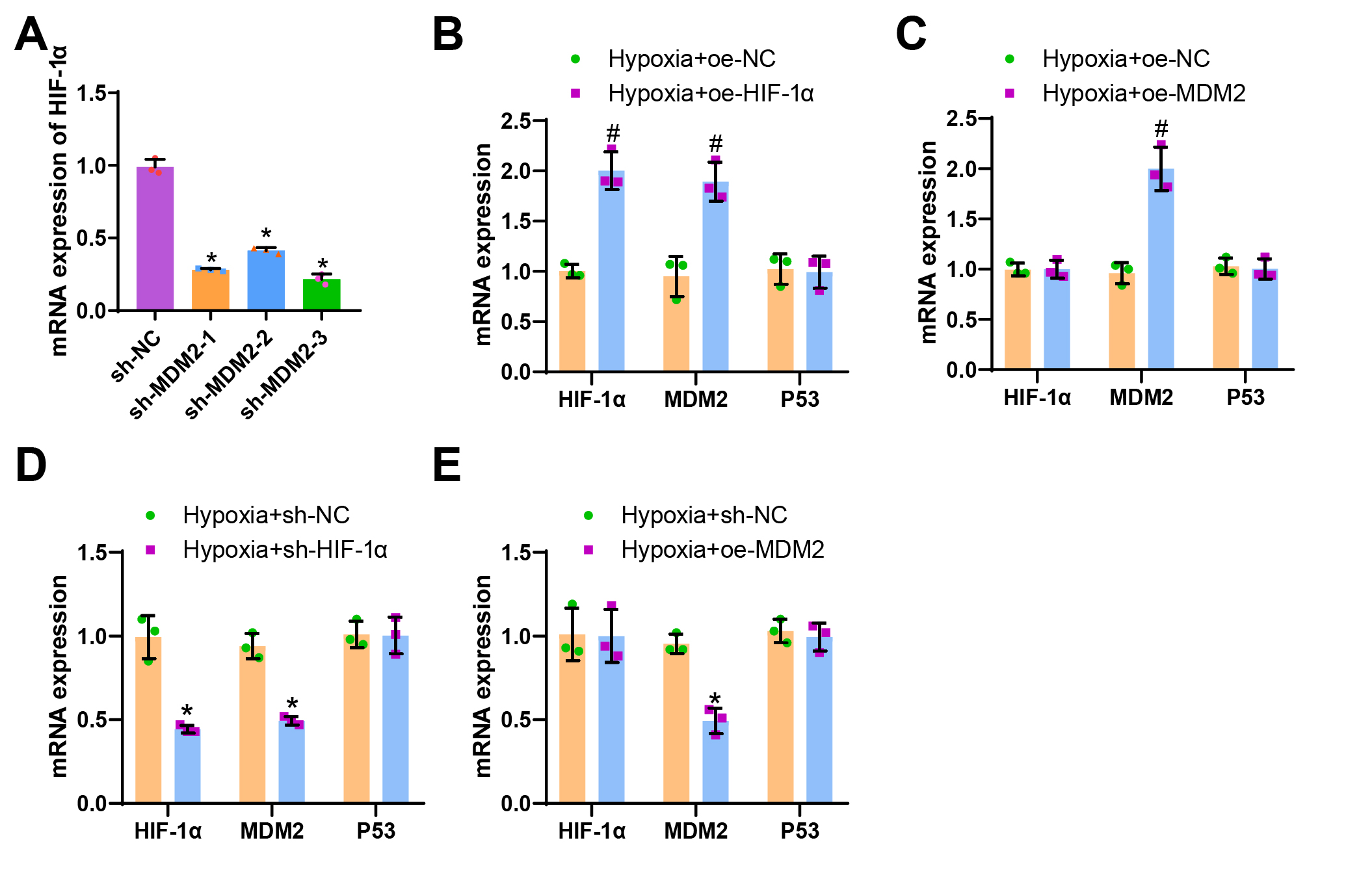

Supplement: Supplementary file 1 — Supplementary Material 1 [file 12931_2024_2869_MOESM1_ESM.jpg]

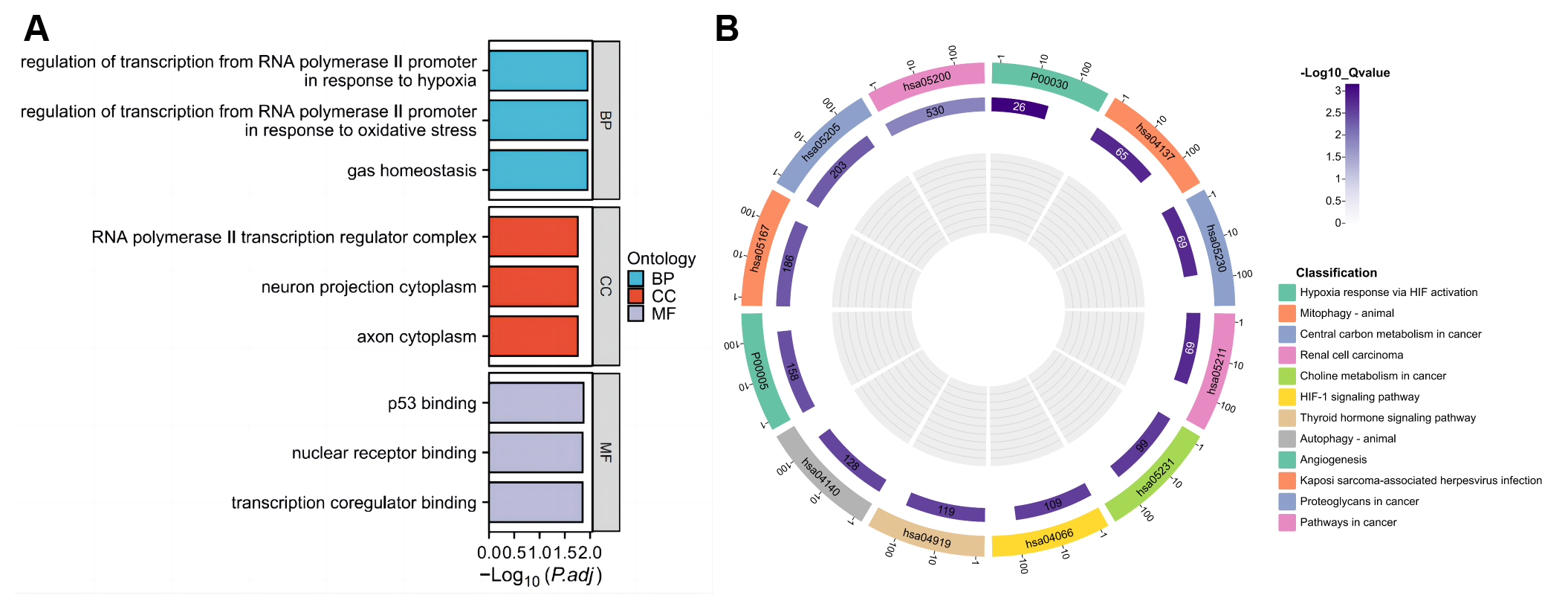

Supplement: Supplementary file 2 — Supplementary Material 2 [file 12931_2024_2869_MOESM2_ESM.jpg]

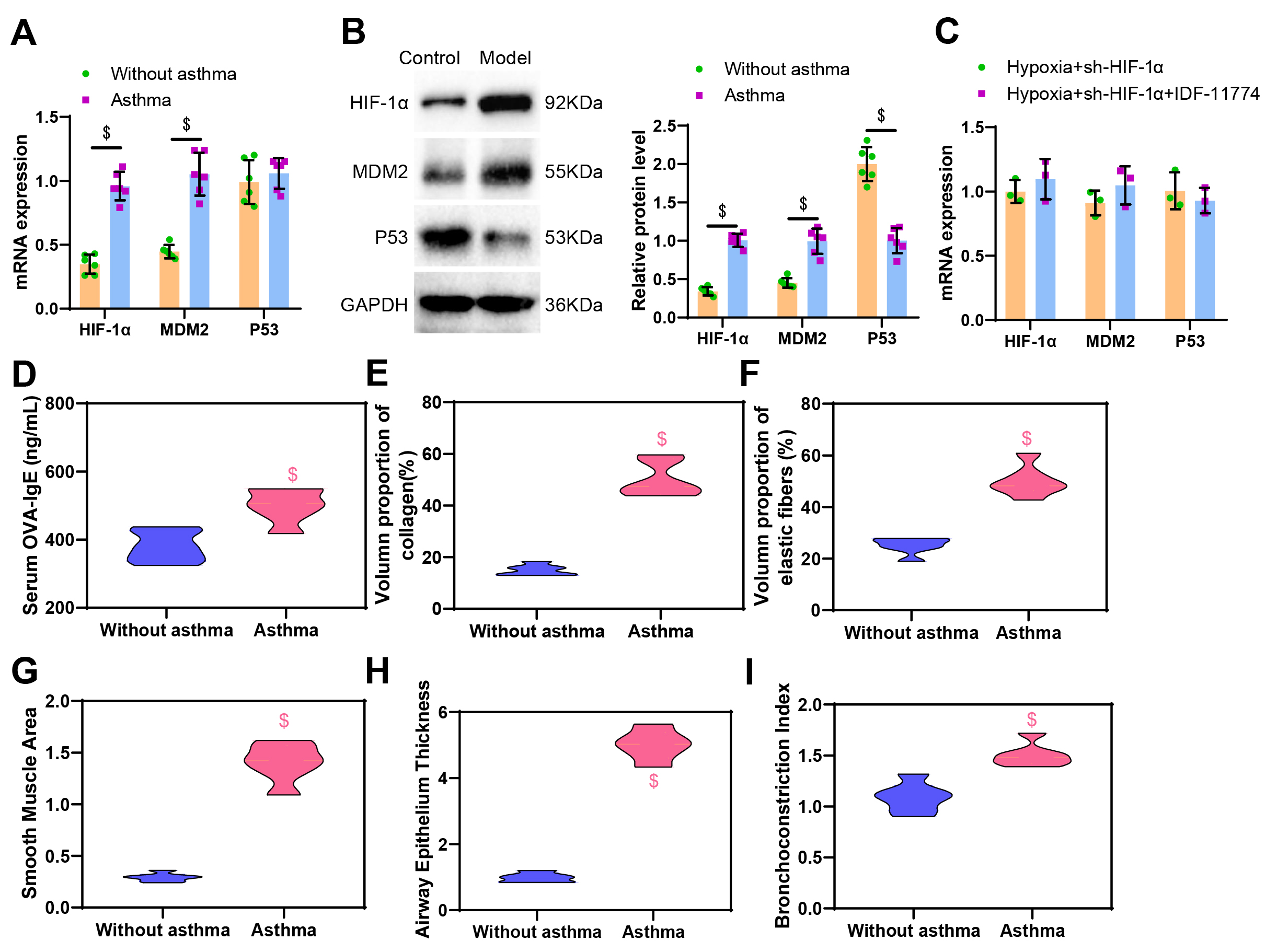

Supplement: Supplementary file 3 — Supplementary Material 3 [file 12931_2024_2869_MOESM3_ESM.jpg]

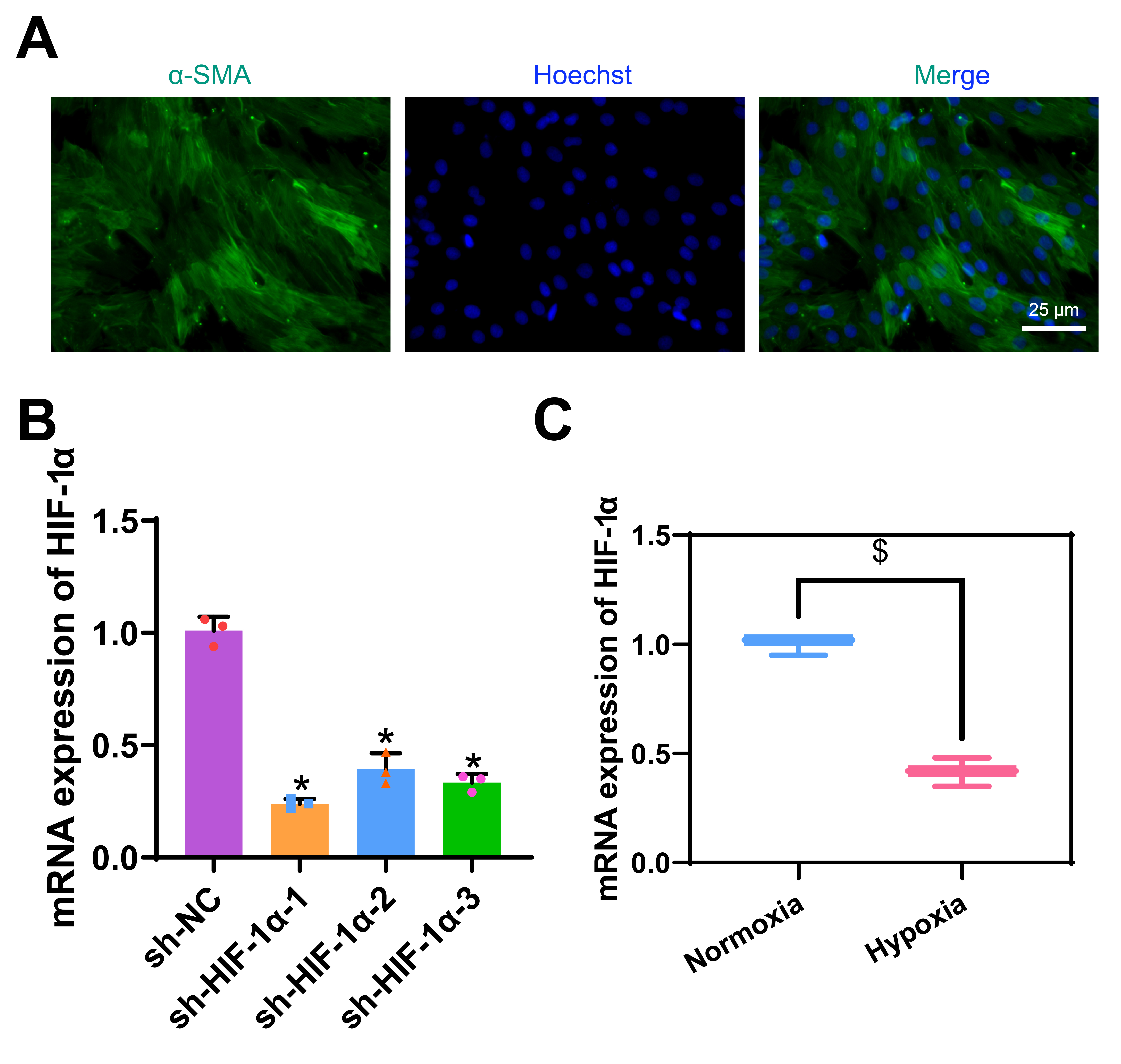

Supplement: Supplementary file 4 — Supplementary Material 4 [file 12931_2024_2869_MOESM4_ESM.jpg]

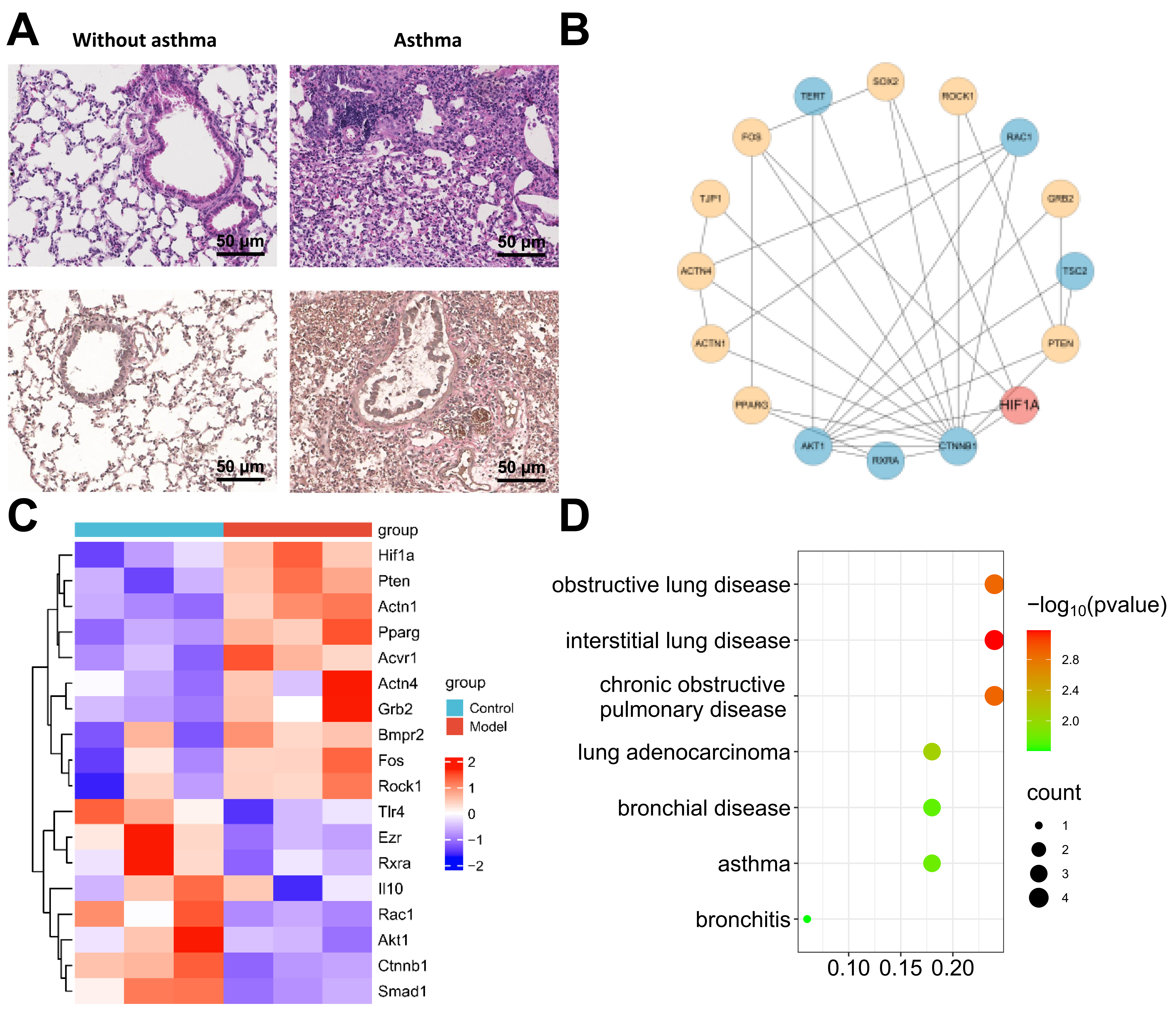

Supplement: Supplementary file 5 — Supplementary Material 5 [file 12931_2024_2869_MOESM5_ESM.jpg]
