# Supplementary material for: Novel hypoxia-induced HIF-1αactivation in asthma pathogenesis
Source: Respir Res. 2024 Jul 25;25:287. doi: 10.1186/s12931-024-02869-0 (PMC11282634; doi:10.1186/s12931-024-02869-0)
Supplement: Supplementary file 6 — Supplementary Material 6 [file 12931_2024_2869_MOESM6_ESM.docx]

**Table S1. shRNA sequences**

| shRNAs | Sequence |
| --- | --- |
| sh-HIF-1α#1 | 5'- TGGATAGCGATATGGTCAATG -3' |
| sh-HIF-1α#2 | 5'- AGTCGACACAGCCTCGATATG -3' |
| sh-HIF-1α#3 | 5'- CCCATTCCTCATCCGTCAAAT -3' |
| sh-MDM2#1 | 5'- CCAATCCAAATGATTGTGCTA -3' |
| sh-MDM2#2 | 5'- CGGAACAAGAGACTCTGGTTA -3' |
| sh-MDM2#3 | 5'- GTGTACCTCATGCAATGAAAT -3' |
| sh-NC | 5'- UGAGUAGACUUAACUUUAA -3' |

**Table S2. RT-qPCR primer sequences**

| Gene | Primer Sequence |
| --- | --- |
| GAPDH (mouse) | F：5'- GGAGAGTGTTTCCTCGTCCC -3' |
|  | R：5'- ATGAAGGGGTCGTTGATGGC -3' |
| HIF-1α (mouse) | F：5'- TTGACAAGCTAGCCGGAGGA -3' |
|  | R：5'- GCGGAGAAAGAGACAAGTCCA -3' |
| P53 (mouse) | F：5'- TTCATTGGGACCATCCTGGC -3' |
|  | R：5'- GGCAGTCATCCAGTCTTCGG -3' |
| MDM2 (mouse) | F：5'- CCCGTGAAGGGTCGGAAGAT -3' |
|  | R：5'- TCCGAAGCTGGAATCTGTGAG -3' |

**F：Forward，R：Reverse**

**Table S3. Details of the first antibody product**

| Name | Cat. | Dilution ratio | Manufacturer | Country |
| --- | --- | --- | --- | --- |
| GAPDH | ab9485 | 1：2500 | Abcam | UK |
| HIF-1α | ab179483 | 1：1000 | Abcam | UK |
| P53 | ab26 | 1-5 µg/mL | Abcam | UK |
| MDM2 | ab259265 | 1：1000 | Abcam | UK |
| IgG | ab6785 | 1：10000 | Abcam | UK |
